# Surveillance and genetic characterization of *Listeria monocytogenes* in the food chain in Montenegro during the period 2014–2022

**DOI:** 10.3389/fmicb.2024.1418333

**Published:** 2024-08-01

**Authors:** Beatriz Daza Prieto, Ariane Pietzka, Aleksandra Martinovic, Werner Ruppitsch, Ivana Zuber Bogdanovic

**Affiliations:** ^1^Institute of Medical Microbiology and Hygiene/National Reference Laboratory for Listeria, Division for Public Health, Austrian Agency for Health and Food Safety, Graz, Austria; ^2^FoodHub – Centre of Excellence for Digitalization of Microbial Food Safety Risk Assessment and Quality Parameters for Accurate Food Authenticity Certification, University of Donja Gorica, Podgorica, Montenegro; ^3^Diagnostic Veterinary Laboratory, Podgorica, Montenegro

**Keywords:** *Listeria monocytogenes*, whole-genome sequencing, food products, antimicrobial resistance genes, Montenegro

## Abstract

**Introduction:**

*Listeria monocytogenes* is an ubiquitous foodborne pathogen that represents a serious threat to public health and the food industry.

**Methods:**

In this study Whole Genome Sequencing (WGS) was used to characterize 160 *L. monocytogenes* isolates obtained from 22,593 different food sources in Montenegro during the years 2014–2022.

**Results:**

Isolates belonged to 21 different clonal complexes (CCs), 22 sequence types (STs) and 73 core genome multilocus sequence types (cgMLST) revealing a high diversity. The most prevalent STs were ST8 (*n* = 29), ST9 (*n* = 31), ST121 (*n* = 19) and ST155 (*n* = 20). All isolates carried virulence genes (VGs), 111 isolates carried mobile genetic elements (MGEs) (ranging from 1 to 7 MGEs) and 101 isolates carried plasmids (ranging from 1 to 3 plasmids). All isolates carried the intrinsic resistance genes *fosX* and *lin*. None of the isolates carried acquired antimicrobial resistance genes (ARGs).

**Discussion/conclusion:**

Continuous monitoring and surveillance of *L. monocytogenes* is needed for improving and ameliorating the public health.

## 1 Introduction

*Listeria monocytogenes* (*L. monocytogenes*) is the etiological agent of human and animal listeriosis (Chlebicz and Slizewska, 2018), with three major invasive clinical presentations in humans: bloodstream infection, infection of the central nervous system, and maternal foetal listeriosis (Hernandez-Milian and Payeras-Cifre, 2014). Strains of *L. monocytogenes* are often isolated from ready-to-eat (RTE) food, including meat, milk, dairy, fish and other seafood, but also ice cream, fresh frozen end processed vegetables, fruits, composite vegetables food and spices (Gambarin et al., 2012; Mateus et al., 2013). This pathogenic bacterium is ubiquitous in different habitats, such as: soil, water, vegetation, animal feed, farms and industrial environment (Kimura, 2006). As a contaminant of food production facilities, *L. monocytogenes* can be found in raw materials, in the environment of the facilities itself, on equipment and the final product. From all these spots*, L. monocytogenes* may spread throughout the facility via aerosols, personnel, food workflows and contaminated contact materials leading to its persistent presence and frequent resistance to hygiene measures for cleaning and disinfection (Zuber et al., 2019). Along with the ability to adapt to different niches and tolerate adverse environmental conditions better than all other non-sporulating bacteria (Liu et al., 2002), the ability of *L. monocytogenes* to form biofilms (Lakicevic and Nastasijevic, 2016) is another property which leads to long-term persistence and repeated cross-contamination of food products (Ferreira et al., 2014).

*Listeria monocytogenes* monitoring programs using molecular sub-typing techniques have been helpful in identifying persistent isolates within whole food chains (Leong et al., 2017). Microbial whole-genome sequencing (WGS) provides identification, characterization and subtyping of a microorganism at the ultimate level of resolution (Ronholm, 2018) making it a superior molecular typing method for monitoring foodborne outbreaks. *L. monocytogenes* is differentiated into 13 serotypes and four phylogenetic lineages of which most human listeriosis cases are associated with *L. monocytogenes* from lineage I, while *L. monocytogenes* from lineage II is commonly found in natural and farm environments (Orsi et al., 2011; Pyz-Lukasik et al., 2022; Benjamin et al., 2023).

The abuse or misuse of antibiotics, in both human and veterinary medicine, has led to an increase in the number of multidrug-resistant strains in recent decades (Maung et al., 2023). Although *L. monocytogenes* is normally susceptible to many antibiotics, some multidrug-resistant strains of *L. monocytogenes* have been isolated from food and the environment (Maung et al., 2023), following its initial discovery in France in 1988 (Poyart-Salmeron et al., 1990). In the food industry, the resistance of *L. monocytogenes* strains to disinfectants complicates the control and eradication of the bacteria in environments where hygiene is crucial (Oloketuyi and Khan, 2017). This poses a significant challenge that many groups address on a daily basis. Even though major advances in this field have been made, it remains crucial to develop safe and effective disinfectants against *L. monocytogenes*.

Virulence factors, encoded by virulence genes (VGs), are the key factor for *L. monocytogenes* to adapt and spread in the environment (Disson et al., 2021; Quereda et al., 2021). *Listeria* Pathogenicity Islands (LIPIs) are regions in the *L. monocytogenes* genomes playing a crucial role in virulence and pathogenicity (Quereda et al., 2021; Wiktorczyk-Kapischke et al., 2023). The four known LIPIs (LIPI-1 – LIPI-4) contain genes involved in various stages of infection like adhesion, invasion, and evasion of the immune response of the host. The key virulence factors encoded by LIPIs are internalins, listeriolysins and phospholipases (Wiktorczyk-Kapischke et al., 2023). LIPI-1 is essential for the intracellular lifecycle of *L. monocytogenes* and encodes the virulence factors InlA, InlB and listeriolysin O (LLO). LIPI-2 enables the movement within host cells and encodes ActA. LIPI-3 is found in certain strains and responsible for the increased virulence (Pyz-Lukasik et al., 2022), carrying *lls* genes (*llsA*, *llsB*, *llsD*, *llsG*, *llsH*, *llsP*, *llsX*, and *llsY*) which encodes *listeriolysin* S (LLS), a hemolytic/cytolytic factor impacting potential virulence. LIPI-4 encodes proteins involved in carbohydrate metabolism contributing to the ability of *L. monocytogenes* to survive and persist in the environment (Wiktorczyk-Kapischke et al., 2023). ST1 and ST4 *L. monocytogenes* have been previously reported of carrying both, LIPI-3 and LIPI-4 and are therefore regarded as hypervirulent clones (Shen et al., 2022). On the other hand, several types of Mobile Genetic Elements (MGEs) like transposons, plasmids and bacteriophages have been identified, that contribute to resistance and enhanced virulence (Parra-Flores et al., 2022).

In this study, we utilized WGS to characterize *L. monocytogenes* isolates collected over an eight-year period (2014–2022) as part of pathogen monitoring in Montenegro’s food industry with the aim to examine similarity and possible common origin sources between isolates. The absence of documented cases of human listeriosis in Montenegro underscores the importance for greater attention regarding this pathogen. Identification and association of identical/similar isolates circulating through the food chain will enhance monitoring efforts significantly. This approach can facilitate the establishment of more effective surveillance programs, helping food operators to successfully meet food safety regulations. Finally, these measures are crucial to improve consumer safety and public health.

## 2 Materials and methods

### 2.1 Origin and sample collection

A total of 22,593 different samples (environmental/food/other origin) were sampled from production facilities, retail shops and control at the borders. The samples were collected by analysing different food products of animal origin, i.e., raw milk and fresh meat during a period of 8 years (2014–2022), through programs of regular monitoring of the presence of *L. monocytogenes* in products on the market (8,698 samples), through programs of official controls (7,373 samples), but also within the framework of regular self-control by producers and food business operators (6,522 samples). The sampling that was done as part of regular monitoring programs as well as official control was carried out in accordance with the sampling plan in compliance with the principles of Commission Regulation (EC) no. 2073/2005 including the sampling of five units of the same production lot. The plan of sampling and testing of products carried out by manufacturers is most often also based on Commission Regulation (EC) no. 2073/2005. Tissue swabs were collected from different surfaces, including those in contact with food in production facilities (*n* = 798), the surface around the facility (*n* = 55) and workers’ hand (*n* = 115). Additionally, samples from raw milk (*n* = 14) and fresh meat (*n* = 34) were collected. For downstream analysis using Whole Genome Sequencing (WGS), one *L. monocytogenes* colony per positive sample was selected.

### 2.2 Cultural detection of *Listeria monocytogenes*

Samples were tested according to the EN ISO 11290-1 standard (ISO, 2022). Thus, samples were enriched (first in half Fraser broth at 30°C for 24 h, then in Fraser broth at 35°C for 48 h) and were then plated on Agar Listeria according to Ottaviani and Agosti (ALOA, Oxoid Ltd., Basingstoke, United Kingdom) and on Palcam agar (Oxoid Ltd., Basingstoke, United Kingdom) and incubated at 37°C for up to 48 h. Colonies which retained typical *L. monocytogenes* appearance were transferred to Tryptone Soya Agar plates supplemented with 0.6% yeast extract (TSYEA, Oxoid, Basingstoke, United Kingdom) and incubated at 37°C for 24 h. All isolates were identified with API Listeria system (BioMérieux SA, France) and all were stored in Brain Heart Infusion Broth (BHI, Oxoid Ltd., Basingstoke, United Kingdom) with 15% glycerol at −80°C. The isolates were refreshed once, every year, and then returned to storage at −80°C.

### 2.3 DNA extraction and whole genome sequencing

For whole genome sequencing (WGS) bacterial isolates were grown on blood agar plates supplemented with 5% sheep blood (COS) (Biomerieux, Vienna, Austria) at 37°C/24 h/aerobiosis. High molecular weight DNA was isolated from overnight cultures using the KingFisher Apex System (Thermofisher, Vienna, Austria) with the MagMAX Viral/Pathogen Ultra Nucleic Acid Isolation Kit (Thermofisher, Vienna, Austria) following the manufacturer’s instruction for Gram-positive bacteria with the following modifications: 2 hours of lysis with 60 μL enzyme mix. DNA was quantified using Dropsense 16 (Trinean NV/SA, Gentbrugge, Belgium) and Qubit Flex Fluorometer (Thermofisher, Vienna, Austria) with 1x dsDNA High Sensitivity (HS) kit (Thermofisher, Vienna, Austria). Genomic libraries were prepared using the DNA Illumina Prep kit (Illumina, San Diego, United States) according to the manufacturer’s instructions. Paired-end sequencing was performed on a NextSeq2000 instrument (Illumina, San Diego, United States) with a read length of 2 × 150 bp (Illumina, San Diego, CA, United States) aiming for a minimum coverage of 30 -fold.

### 2.4 Sequence data analysis

Raw reads were *de novo* assembled using SPAdes (version 3.11.1) (Bankevich et al., 2012). Contigs were filtered for a minimum coverage of 5 and a minimum length of 200 base pairs using SeqSphere+ software v8.5.1 (Ridom, Münster, Germany).

Subtyping of isolates was conducted in SeqSphere+ software v8.5.1 with classical multilocus sequence typing (MLST) based on the seven housekeeping genes as described by Salcedo et al. (2003), Clonal Complex (CC) (Ragon et al., 2008), core genome (cg)MLST (Werner et al., 2015) using a cluster threshold of 10 allelic differences.

Information on serogroups was extracted from genome data based on the 5-plex PCR (*lmo118*, *lmo0737*, *ORF2110*, *ORF2829* and *prs*) as described (Michel et al., 2004; Lee et al., 2012).

NCBI AMRFinder+ v3.11.2. was used with the EXACT method at 100% setting together with the BLAST alignment at >90% of protein sequences against the AMRFinder+ database to detect antimicrobial resistance genes (ARG) (Michael et al., 2019). The Virulence Factor Database (VFDB) (http://www.mgc.ac.cn/cgi-bin/VFs/genus.cgi?Genus=Listeria; accessed on 6th June 2023) was used to detect VGs (Liu et al., 2022). Thresholds were set for the target scanning procedure to ≥85% identity with the reference sequence and ≥ 99% with the aligned reference sequence. Pathogenicity islands LIPI-3 and LIPI-4 were extracted from genome data. The CGE Mobile Element Finder v1.0.5. was used with the <90% identity and > 95% alignment method to detect Mobile Genetic Elements (MGE) (Johansson et al., 2021). Chromosome & Plasmid Finder v1.0. through MOB-suite v3.1.4. available in SeqSphere+ v8.5.1. was used with the Mash Neighbor Distance >0.06 to detect plasmids (Robertson and Nash, 2018). Isolates of the most prevalent STs of *L. monocytogenes* of our study (ST8, ST9, ST121, ST155, ST204) were compared with those from pubMLST database^1^ and the Austrian Reference Laboratory for Listeria, which currently includes more than 20,000 genomes.

## 3 Results

### 3.1 Detection of *Listeria monocytogenes*

Out of 22,593 samples tested, *L. monocytogenes* was detected in 160 (0.7%) of them. From each positive sample, a single colony of *L. monocytogenes* was isolated, resulting in a collection of isolates consisting of 147 isolates from Montenegro and 13 isolates from food products imported from other European countries. During 8 years of monitoring the presence of *L. monocytogenes* in the food chain, a total of 986 environmental swabs, 48 raw material samples and 21,559 RTE food samples were examined. From all 147 isolates with Montenegrin origin, 131 isolates were from food products from 19 different industries, five isolates were isolated from small farms (households) and 11 *L. monocytogenes* isolates were isolated from unknown companies from Montenegro between 2014 and 2015. In addition, 13 isolates were obtained from food imported from different European countries: France = 1, Serbia = 2, Italy = 9 and Bosnia and Hercegovina = 1 (Tables 1, 2). From the total of isolates included in the study two *L. monocytogenes* isolates were obtained from environmental swabs, eight *L. monocytogenes* isolates originated from raw material and 150 *L. monocytogenes* isolates originated from food. 80% of the isolates originated from meat (pork, beef, chicken, and mixed meat), 16% from dairy products (mozzarella, cow cheese, butter, fresh milk), 0.7%, from ready-to-eat fish, 0.7% from frozen vegetables, 0.7% from food contact surfaces in production plants, and 1.8% were of unknown origin.

**Table 1:** Information on diversity of industries/producers, years, and sequence types (STs) in the *L. monocytogenes* isolates included in this study.

Industry (No. of isolates)	Years	Sequence type	Prevalence per industry (%)
Meat industry 1 (23)	2015 2016 2017 2018 2019 2021 2022	ST9 ST9, ST121 ST8, ST9 ST4, ST9 ST7, ST8 ST8, ST9, ST121 ST9	ST4 (4%) ST7 (4%) ST8 (13%) ST9 (48%) ST121 (31%)
Meat industry 2 (34)	2016 2019 2020 2021 2022	ST8, ST9, ST37, ST121 ST31, ST101, ST580 ST8, ST121, ST155, ST451, ST580 ST8 ST8	ST8 (50%) ST9 (10%) ST31 (6%) ST37 (3%) ST101 (6%) ST121 (6%) ST155 (6%) ST451 (3%) ST580 (10%)
Meat industry 3 (9)	2017 2020 2021	ST9, ST736 ST8 ST736	ST8 (22%) ST9 (22%) ST736 (56%)
Meat industry 4 (1)	2021	ST1	ST1 (100%)
Meat industry 5 (6)	2019 2020	ST9, ST121, ST321 ST9	ST9 (50%) ST121 (33%) ST321 (17%)
Meat industry 6 (12)	2015 2016 2017 2019 2020	ST9 ST8, ST9, ST451 ST121 ST9 ST8, ST37, ST489	ST8 (18%) ST9 (37%) ST37 (9%) ST121 (9%) ST451 (9%) ST489 (17%)
Meat industry 7 (1)	2019	ST155	ST155 (100%)
Meat industry 8 (4)	2015 2019	ST7, ST9 ST8	ST7 (25%) ST9 (50%) ST9 (25%)
Meat industry 9 (10)	2016 2019	ST1, ST321 ST8, ST121, ST321	ST1 (10%) ST8 (30%) ST121 (40%) ST321 (20%)
Meat industry 10 (2)	2018 2019	ST121 ST37	ST37 (50%) ST121 (50%)
Meat industry 12 (1)	2016	ST3	ST3 (100%)
Meat industry 13 (2)	2016 2017	ST8 ST121	ST8 (50%) ST121 (50%)
Meat industry 15 (3)	2016	ST8, ST37, ST451	ST8 (34%) ST37 (33%) ST451 (33%)
Meat industry 16 (1)	2016	ST8	ST8 (100%)
Meat industry 17 (3)	2015	ST2	ST2 (100%)
Milk industry 1 (2)	2020	ST101	ST101 (100%)
Milk industry 2 (9)	2016 2017 2018	ST8, ST204 ST204 ST37, ST204	ST8 (11%) ST37 (11%) ST204 (78%)
Milk industry 3 (7)	2016 2017 2018	ST155 ST155 ST155	ST155 (100%)
Milk industry 4 (1)	2017	ST7	ST7 (100%)
Small farms (5)	2017 2018 2019	ST1 ST7, ST121, ST204 ST26	ST1 (20%) ST7 (20%) ST26 (20%) ST121(20%) ST204 (20%)
Unknown industries (11)	2014 2015	ST9, ST101, ST517, ST736 ST1, ST2, ST124, ST451	ST1 (9%) ST2 (9%) ST9 (37%) ST101 (9%) ST124 (9%) ST451 (9%) ST517 (9%) ST736 (9%)
Imported (13)			
IMP 1 IMP 2 IMP 3 and IMP 4 IMP 5	2017 Bosnia 2020 France 2018–2019 Serbia 2022 Italy	ST7 ST37 ST14, ST155 ST155	ST7 (8%) ST14 (8%) ST37 (8%) ST155 (77%)

**Table 2:** Information on *L. monocytogenes* isolates belonging to different clusters included in this study.

Cluster	No. of isolates	ST	CT	Source	Industry	Year	Country
1	18	8	69	Fermented beef sausage	Meat IND 1	2017	Montenegro
			69, 73, 1358, 5250, 5503, 16828	Smoked pork sausage, prosciutto, pancetta	Meat IND 2	2016–2022	Montenegro
			16783	Prosciutto	Meat IND 6	2020	Montenegro
			16783	Pancetta	Meat IND 13	2016	Montenegro
2	11	9	16782	Fermented beef sausage	Meat IND 1	2016–2022	Montenegro
3	9	155	2760	Chicken hot dog	IMP 5	2022	Italy
4	8	204	16788	Mozzarella	Milk IND 2	2016–2018	Montenegro
5	7	121	2134, 16793	Fermented beef sausage	Meat IND 1	2016–2021	Montenegro
6	7	155	1234, 8675, 16789	Cow cheese, butter	Milk IND 3	2016–2018	Montenegro
7	7	8	5503	Smoked pork sausage	Meat IND 3	2020	Montenegro
				Fresh pork meat	Meat IND 8	2019	
				Smoked pork sausage	Meat IND 9	2019	
8	6	736	16577	Pork meat	Unknown	2014	Montenegro
					Meat IND 3	2017–2021	
9	5	9	354, 3779	Smoked pork sausage, pancetta	Meat IND 6	2015–2019	Montenegro
			1691	Smoked pork sausage	Meat IND 8	2015	
10	5	9	17054	Pancetta	Meat IND 2	2016	Montenegro
			1698	Smoked pork sausage	Meat IND 8	2015	
			1704	Fresh pork meat, unknown	Unknown	2014	
11	5	580	15473	Pork meat	Meat IND 2	2019–2020	Montenegro
12	5	101	3186	Prosciutto	Meat IND 2	2019	Montenegro
				Cow cheese, fresh milk	Milk IND 1	2020	
				Smoked pork sausage	Unknown	2014	
13	4	9	358	Smoked pork sausage	Meat IND 2	2016	Montenegro
				Smoked pork sausage	Meat IND 3	2017	
14	4	2	8598	Smoked pork sausage, unknown	Meat IND 17	2015	Montenegro
			8598	Prosciutto	Unknown	2015	
15	4	121	8919	Smoked pork sausage	Meat IND 2	2016	Montenegro
				Fresh bacon, smoked pork sausage	Meat IND 9	2019	
16	3	321	197	Mixed frozen meat	Meat IND 5	2019	Montenegro
				Pork meat	Meat IND 9	2016, 2019	
17	2	155	3856	Smoked pork sausage	Meat IND 2	2020	Montenegro
18	2	121	2907, 16784	Pancetta, prosciutto	Meat IND 5	2019	Montenegro
19	2	121	5750	Smoked pork sausage	Meat IND 10	2018	Montenegro
					Meat IND 13	2017	
20	2	9	285	Pancetta	Meat IND 5	2019	Montenegro
21	2	7	16794	Cow meat	Milk industry 4	2017	Montenegro
				Sour cream	IMP 1	2017	Bosnia and Hercegovina
22	2	489	16785	Pork meat	Meat industry 6	2020	Montenegro
23	2	31	6240	Pork meat	Meat industry 2	2019	Montenegro

### 3.2 Whole genome sequence based subtyping, description of clusters, and description of serogroups

**Figure 1:**
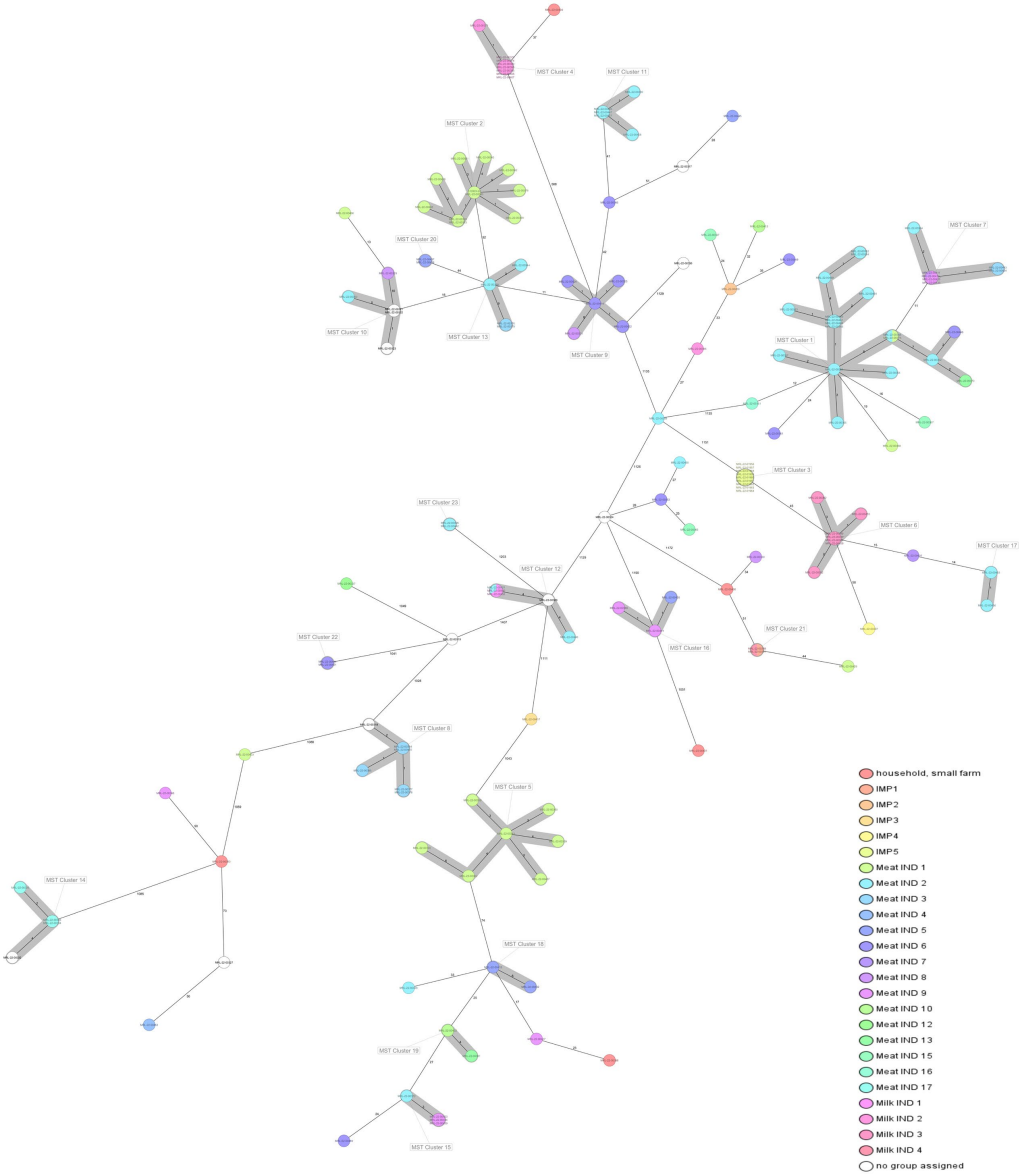
Minimum spanning tree (MST) of 160 *L. monocytogenes* isolates included in the study based on the cgMLST defined by Ruppitsch et al. (2015). Cluster definition was set in 10 allelic differences. Isolates are coloured by company/industry. Information in every isolate: ST, alias, year of isolation.

WGS based characterization of the 160 *L. monocytogenes* isolates revealed a high diversity. Isolates belonged to four different serogroups [IIa (*n* = 105), IIb (*n* = 10), IIc (*n* = 36) and IVb (*n* = 9)], 21 different clonal complexes (CCs), 22 different sequence types (STs) and 73 cgMLST complex types (CTs). One hundred twenty-two isolates belonged to a total of 23 cluster and 38 isolates were singletons (Figure 1; Table 2). Number of isolates per industry ranged from one isolate to 34 isolates (Table 1).

*Listeria monocytogenes* isolates belonging to serogroup IIa encompassed 13 different STs (ST7, ST8, ST14, ST26, ST31, ST37, ST101, ST121, ST124, ST155, ST204, ST321, and ST451), isolates belonging to serogroup IIb encompassed 4 different STs (ST3, ST489, ST517, and ST736), isolates belonging to serogroup IIc encompassed 2 different STs (ST9 and ST580), and *L. monocytogenes* isolates belonging to serogroup IVb encompassed 3 different STs (ST1, ST2, and ST4).

**Figure 2:**
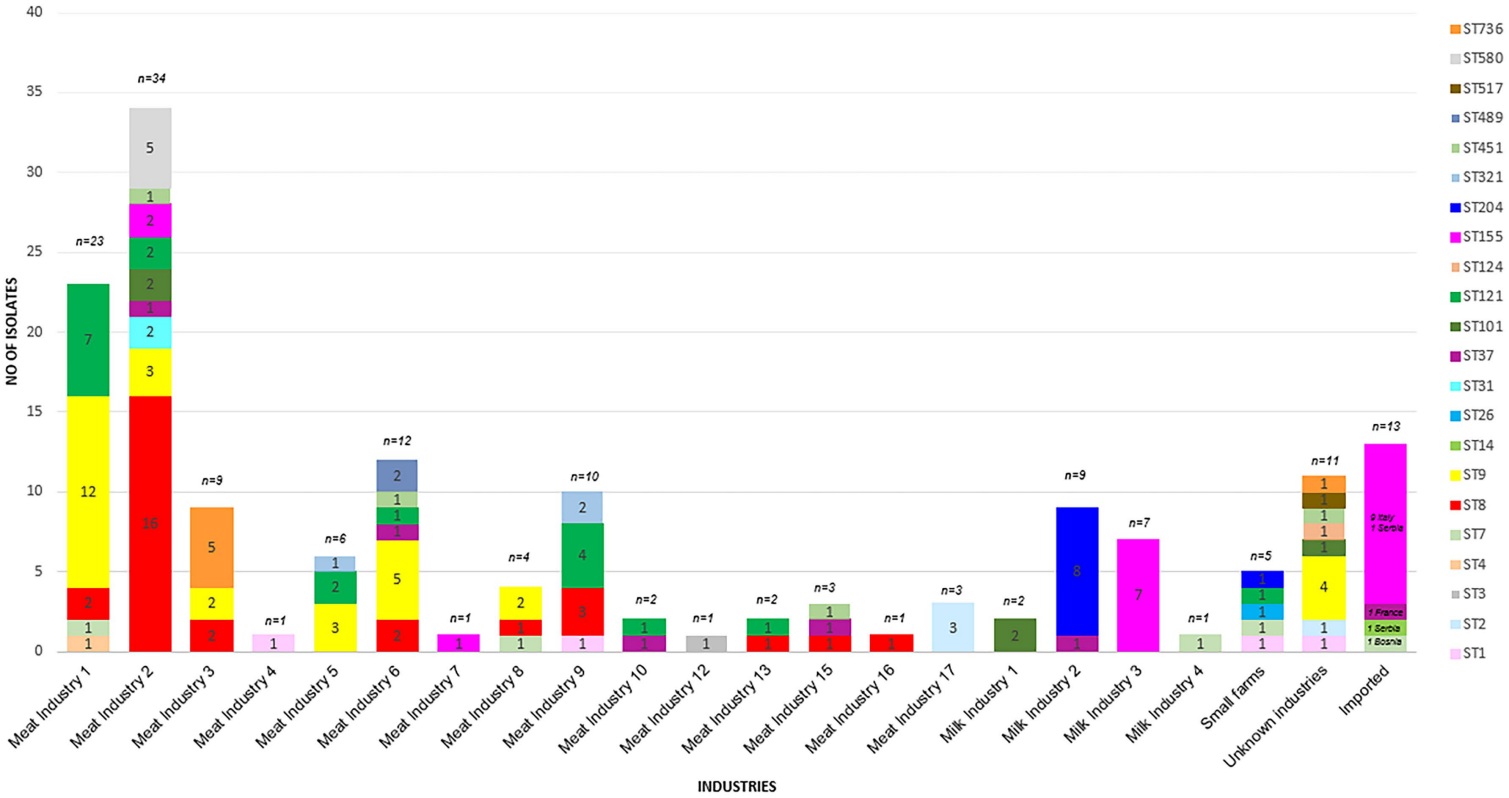
Prevalence of each ST sorted by industry.

The most prevalent STs were ST8 (*n* = 29), ST9 (*n* = 31), ST121 (*n* = 19), ST155 (*n* = 20, including 9 isolates assigned to the Italian hotdog outbreak), and ST204 (*n* = 9) from the period 2015–2020 (Figure 2). The industries with the highest prevalence of isolates were Meat IND 1 (*n* = 23), Meat IND 2 (*n* = 34) and Meat IND 6 (*n* = 12).

**Figure 3:**
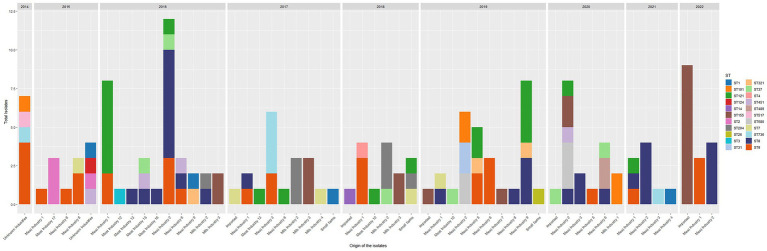
ST sorted by industry and year.

*L. monocytogenes* isolates from Meat IND 1 (*n* = 23) encompassed five different STs (Table 1; Figures 2, 3). ST9 was the most prevalent with twelve isolates (52%) being detected every year from 2015–2022 (except 2019). All isolates belonged to cluster 2 (Figure 1). ST121 was the second most prevalent with seven isolates (31%). ST121 was the most prevalent type in the year 2016.

*Listeria monocytogenes* isolates from Meat IND 2 (*n* = 34) encompassed nine different STs (Table 1; Figures 2, 3). ST8 was the most prevalent with sixteen isolates (50%). ST8 was detected every year from 2016 to 2022 (except in 2019). All isolates belonged to cluster 1 (Figure 1).

*L. monocytogenes* isolates from Meat IND 6 (*n* = 12) encompassed six different STs (Table 1; Figures 2, 3). ST9 was the most prevalent with five isolates (37%) detected in 2015, 2016 and 2019. Four isolates belonged to cluster 9. One ST9 isolate differed by 1,129 alleles from cluster 9 (Figure 1).

*Listeria monocytogenes* isolates from Milk IND 1, 2, 3 and 4 encompassed five different STs (Table 1; Figures 2, 3) from years 2016–2020. ST155 was the most prevalent with seven isolates (35% of the ST155) in Milk IND 3. All isolates belonged to cluster 6 (Figure 1). ST204 was the most prevalent with nine isolates (100% of the total ST204) in Milk IND 2 and one small farm. All isolates belonged to cluster 4 (Figure 1).

#### 3.2.1 *Listeria monocytogenes* ST8 (*n* = 29)

Twenty-nine *L. monocytogenes* ST8 isolates (18% of the total) were obtained from ten industries in Montenegro during the period 2016–2022. ST8 isolates were the most prevalent in Meat IND 2 (*n* = 16, 55,2% of ST8), Meat IND 13 (*n* = 1, 50%) and Meat IND 16 (*n* = 1, 100%). *L. monocytogenes* ST8 isolates were distributed to cluster 1 (*n* = 18), cluster 7 (*n* = 7) and four singletons. Cluster 1 comprised one isolate from Meat IND 1 (3.4% of the ST8), fifteen isolates from Meat IND 2 (48%), one isolate from Meat IND 6 (3.4%) and one isolate from Meat IND 13 (3.4%) with a maximum of eight allelic differences within the cluster (Figure 1). Cluster 7 comprised seven isolates differing by a maximum of three alleles. One isolate was obtained from Meat IND 2 (3.4%), two isolates from Meat IND 3 (7%), one isolate from Meat IND 8 (3.4%) and three isolates from Meat IND 9 (10%) (Figure 1). Cluster 1 and cluster 7 isolates differed by a minimum of 11 allelic differences (Figure 1). The four ST8 singletons were isolated from Meat IND 1 from 2021, Meat IND 6 from 2016, Meat IND 15 from 2016 and Meat IND 16 from 2016 (Figure 1; Supplementary Figure S1). They differed from each other by a minimum of 20 and a maximum of 35 alleles and from cluster 1 and cluster 7 isolates by a minimum of 12 and 19 alleles, respectively.

ST8 isolates from Meat IND 2 (cluster 1) were differing by 12 alleles from a salmon isolate from Poland (CC8/ST8/CT1269) (accession no. SRR975366) (Moura et al., 2016) and by 18 alleles from a human isolate from France (CC8/ST8/CT2873) (accession no. ERR1100940) (Maury et al., 2016; Moura et al., 2016).

#### 3.2.2 *Listeria monocytogenes* ST9 (*n* = 31)

Thirty-one *L. monocytogenes* ST9 isolates (19% of the total) were obtained from ten different industries in Montenegro from the period 2014 to 2022. ST9 isolates were the most prevalent type in Meat IND 1 (*n* = 12, representing the 38% of ST9), Meat IND 5 (*n* = 3, 10%), Meat IND 6 (*n* = 5, 16%), and Meat IND 8 (*n* = 2, 6%) (Table 1; Figure 2). *L. monocytogenes* ST9 isolates were distributed to five clusters (cluster 2, 9, 10, 13 and 20) and four isolates were singletons. Cluster 2 isolates (*n* = 11, 35.48% of ST9) were obtained from beef meat from Meat IND 1 from 2015 to 2022, whereas isolates from the other clusters were derived from pork meat. Cluster 9 isolates (*n* = 5, 16.12% of ST9) were obtained from Meat IND 6 and Meat IND 8, with a maximum of eight allelic difference. Isolates within Cluster 10 (*n* = 5, 16.12% of ST9) were obtained from Meat IND2 (*n* = 1, 3.22%), Meat IND 8 (*n* = 1, 3.22%) and from and unknown industries (*n* = 3, 9.67%), with a maximum of ten allelic differences within the cluster. Cluster 13 isolates (*n* = 4; 12.90% of ST9) were obtained from Meat IND 2 and Meat IND 3 with a maximum difference of nine alleles. Isolates in Cluster 20 (*n* = 2, 6.45% of ST9) derived from Meat IND 5 showed no allelic difference in their core genome.

A ST9 singleton (MRL-22-00345) from Meat IND 6 differed by 18 and 23 alleles from two human isolates from France, (CC9/ST9/CT7213) (accession no. ERR1100975) and (CC9/ST9/CT7272) (accession no. ERR1100950) (Maury et al., 2016; Moura et al., 2016), respectively. ST9 isolates from Meat IND 6 (cluster 9) differed by 50 alleles from a human isolate from France (CC9/ST9/CT7208) (accession no. ERR1100972) (Maury et al., 2016; Moura et al., 2016). ST9 isolates from Meat IND 2 (cluster 13) differed by 25 alleles from a human isolate from France (CC9/ST9/CT7211) (accession no. ERR1100973) (Maury et al., 2016; Moura et al., 2016). Strains with the same cgMLST profile (CC9/ST9/CT354) and (CC9/ST9/CT3779), mainly obtained from meat samples, were found in the database of the Austrian National Reference Laboratory (NRL) for Listeria (personal communication).

#### 3.2.3 *Listeria monocytogenes* ST121 (*n* = 19)

Nineteen *L. monocytogenes* ST121 isolates (12% of the total) were obtained from seven industries and one small farm from 2016 to 2021. *L. monocytogenes* ST121 were distributed to four clusters (cluster 5, 15, 18 and 19) and four singletons. Cluster 5 (*n* = 7, representing 37% of ST121) comprised isolates obtained from Meat IND 1 from the years 2016 (*n* = 6) and 2021 (*n* = 1) differing by a maximum of six alleles (Table 1; Figure 2). Cluster 15 (*n* = 4; representing 21% of ST121) isolates were obtained from Meat IND 2 in 2016 (*n* = 1) and from Meat IND 9 in 2019 (*n* = 3, representing 16% of ST121) with a maximum of three allelic differences. Cluster 18 (*n* = 2) isolates were obtained from Meat IND 5 in 2019. Cluster 19 (*n* = 2; representing 10% of ST121) isolates were obtained from Meat IND 10 in 2018 and Meat IND 13 in 2019. Four ST121 singletons were obtained from three industries (Meat IND 2, 6, 9) and a small farm differing by 23 to 61 alleles from each other and by 17 to 51 alleles from the clusters (Figure 1; Supplementary Figure S2).

ST121 isolates from Meat IND 5 (cluster 17) differed by 9, 21 and 68 alleles from French isolates originating from human and food-associated sources (CC121/ST121/CT2907) (accession no. 1100947), (CC121/ST121/CT7267) (accession no. ERR1100919) and (CC121/ST121/CT7212) (accession no. 1100974), respectively, (Maury et al., 2016; Moura et al., 2016).

#### 3.2.4 *Listeria monocytogenes* ST155 (*n* = 20)

Twenty *L. monocytogenes* isolates ST155 were obtained from three industries in Montenegro, one industry from Serbia and one from Italy from 2016 to 2022. ST155 isolates were distributed to three clusters (cluster 3, 6 and 17) and two isolates were singletons. Cluster 3 (*n* = 9, representing 45% of ST155) comprised identical isolates obtained from chicken sausages from industry IMP 5 from Italy in 2022. Cluster 6 (*n* = 7, representing 35% of ST155) isolates were obtained from Milk IND 3 from 2016 and 2018 differing by a maximum of three alleles. Cluster 17 (*n* = 2; 10% of ST155) isolates were obtained from pork meat from Meat IND 2 in 2020 showing one allelic difference. Two singletons were obtained from industry IMP 4 (Serbia, 2018) and beef meat from meat IND 7 in 2019 differing by a minimum of 62 alleles from each other (Figure 1; Supplementary Figure S3).

ST155 isolates from Milk IND 3 (cluster 6) differed by 32, 42 and 51 alleles from one isolate from Finland (CC155/ST155/CT15) (accession no. NC_017547) (den Bakker et al., 2013; Maury et al., 2016), two isolates from France [one from human (CC155/ST155/CT2885) (accession no. ERR1100937) (Maury et al., 2016; Moura et al., 2016) and one from salmon (CC155/ST155/CT7342) (accession no. ERR10849967) (Moura et al., 2024)].

A clinical isolate from Serbia (unpublished, personal communication) from the year 2015 (CC155/ST155/CT2760) available through the NRL for Listeria database differed by 5 alleles from cluster 3 food isolates (Italian outbreak; EpiPulse ID: 2022-FWD-00053). In addition, nine clinical isolates from Serbia and eight isolates from Austria isolated from fish (CC155/ST155/CT2842) from the NRL for Listeria database clustered with a singleton from our study (MRL-22-00397, CC155/ST155/CT2842), which was obtained from smoked salmon (personal communication).

#### 3.2.5 *Listeria monocytogenes* ST204 (*n* = 9)

A total of nine *L. monocytogenes* ST204 isolates were obtained from Milk IND 2 (*n* = 8, representing 88% of ST204) and one small farm in Montenegro (*n* = 1, 12%). All Milk IND 2 isolates belonged to cluster 4 differing by a maximum of one allele within the cluster, whereas the small farm isolate differed by a minimum of 37 alleles from cluster 4 (Figure 1; Supplementary Figure S4).

#### 3.2.6 Other *Listeria monocytogenes* STs

Six ST736 isolates (3.75% of the total) obtained from Meat IND 3 from 2017 to 2021 and from an unknown industry in 2014 belonged to cluster 8. Five ST580 isolates (3.125% of the total) from Meat IND 2 belonged to cluster 11. Five ST101 isolates (3.125% of the total of isolates) obtained from Milk IND 1 in 2020, Meat IND 2 in 2019, and an unknown industry in 2014 a belonged to cluster 12 (Table 2; Figure 1). Twenty-three isolates were singletons obtained from different sources, industries, and years (Table 3). Singletons belonging to ST1 (*n* = 4, 2.5% of the total), ST37 (*n* = 6, 3.75%) and ST451 (*n* = 4, 2.5%) were the most prevalent and were obtained from different sources and from different industries (Table 3).

**Table 3:** Information on *L. monocytogenes* singletons isolates included in this study.

Sample ID	ST	CT	Source	Industry	Year	Country
MRL-22-00393 MRL-22-00463 MRL-22-00327 MRL-22-00368	1	16797 16036 16803 17053	Cow cheese Pork meat Pork meat Pork meat	Small farm Meat IND 4 Unknown Meat IND 9	2017 2021 2015 2016	Montenegro Montenegro Montenegro Montenegro
MRL-22-00337	3	16787	Pork meat	Meat IND 12	2016	Montenegro
MRL-22-00403	4	17052	Beef meat	Meat IND 1	2018	Montenegro
MRL-22-00330 MRL-22-00429 MRL-22-00400	7	7998 16798 3879	Pork meat Beef meat Cow cheese	Meat IND 8 Meat IND 1 Small farm	2015 2019 2018	Montenegro Montenegro Montenegro
MRL-22-00351 MRL-22-00361 MRL-22-00468 MRL-22-00367	8	4172 8350 16829 8506	Smoked pork sausage Smoked pork sausage Fermented beef sausage Smoked pork sausage	Meat IND 16 Meat IND 6 Meat IND 1 Meat IND 15	2016 2016 2021 2016	Montenegro Montenegro Montenegro Montenegro
MRL-22-00317 MRL-22-00445 MRL-22-00345 MRL-22-00406	9	16576 5099 15203 3755	Smoked pork sausage Smoked pork sausage Smoked pork sausage Smoked pork sausage	Unknown Meat IND 5 Meat IND 6 Meat IND 1	2014 2020 2016 2018	Montenegro Montenegro Montenegro Montenegro
MRL-22-00417	14	16799	Beef meat	IMP 3	2019	Serbia
MRL-22-00431	26	16781	Cow cheese	Small farm	2019	Montenegro
MRL-22-00449 MRL-22-00347 MRL-22-00396 MRL-22-00413 MRL-22-00459 MRL-22-00358	37	5456 17051 16805 14979 3311 16792	Pork meat Pork meat Cow cheese Pork meat Mixed frozen vegetables Pork meat	Meat IND 6 Meat IND 15 Milk IND 2 Meat IND 10 IMP 2 Meat IND 2	2020 2016 2018 2019 2020 2016	Montenegro Montenegro Montenegro Montenegro France Montenegro
MRL-22-00427 MRL-22-00398 MRL-22-00448 MRL-22-00389	121	16804 17050 16798 16801	PVC swab Cow cheese Smoked pork sausage Smoked pork sausage	Meat IND 9 Small farm Meat IND 2 Meat IND 6	2019 2018 2020 2017	Montenegro Montenegro Montenegro Montenegro
MRL-22-00336	124	16790	Pork meat	Unknown	2015	Montenegro
MRL-22-00397 MRL-22-00416	155	2842 5044	Smoked salmon Fresh beef meat	IMP 4 Meat IND 7	2018 2019	Serbia Montenegro
MRL-22-00404	204	16802	Cow cheese	Small farm	2018	Montenegro
MRL-22-00324 MRL-22-00455 MRL-22-00353 MRL-22-00365	451	16578 3999 3790 16800	Ham Pork meat Pork meat Pork meat	Unknown Meat IND 2 Meat IND 6 Meat IND 15	2015 2020 2016 2016	Montenegro Montenegro Montenegro
MRL-22-00319	517	5481	Pork meat	Unknown	2014	Montenegro

Some singletons obtained from pork meat with less prevalent STs from our study showed a close genetic relationship with strains from the Austrian NRL for Listeria database (unpublished, personal communication). Singleton CC1/ST1/CT16036 (MRL-22-00463) was identical to two Austrian strains obtained from ice-cream. Singleton CC7/ST7/CT7998 (MRL-22-00330) differed by 4 and 9 alleles, from two Austrian isolates obtained from sausage and sheep meat. Singleton CC37/ST37/CT5456 (MRL-22-00449) differed by one allele to four Austrian strains obtained from meat and meat products. Singleton CC11/ST451/CT3790 (MRL-22-00353) clustered with 40 Austrian strains which were obtained from meat and meat products. Singleton CC517/ST517/CT5481 (MRL-22-00319) clustered with 44 Austrian isolates, which were obtained from meat and meat products.

Cluster 16 isolates (CC321/ST321/CT197) from our study differed by nine alleles from a clinical isolate (accession no SRR13744450) from Queens, United States of America.

Detailed information about isolates included in this study are available in the supplements (Supplementary Table S1).

### 3.3 Mobile genetic elements and plasmids

One hundred eleven out of 160 isolates carried at least one MGE. Nine isolates (5.6% of the total) carried over five MGEs. The most prevalent MGE was the unit transposon *Tn5422*, which was found in eighty-four isolates (52.5% of the total) followed by the insertion sequences *ISLmo1* and *ISLmo9* found in forty-three (26.8% of the total) and forty-seven (29.3% of the total) isolates, respectively (Supplementary Table S1). All *L. monocytogenes* ST8, ST9 and ST121 isolates carried MGE *Tn5422. ISLmo1*, *ISLmo7*, *ISLmo8*, and *ISLmo9* were present in 32, 39, 39, and 55% of *L. monocytogenes* ST9, respectively. All ST204 and two ST155 isolates carried *ISLmo1*. The remaining eighteen ST155 isolates and ST1, ST2, ST4, ST7, ST451, ST489, ST517, ST736 isolates carried no MGEs. ST3, ST9, ST31, and ST580 carried 5–7 MGEs (Supplementary Table S1).

One hundred one out of 160 *L. monocytogenes* isolates carried at least one plasmid. The plasmid sizes ranged from 25.6–432.1 kb. All *L. monocytogenes* isolates belonging to ST3, ST8, ST9, ST31, ST121, ST321, ST517 and ST580 carried plasmids (Supplementary Table S1; Supplementary Figure S5). *L. monocytogenes* isolates belonging to those ST profiles were obtained from pork or beef sausages from different meat industries. Seven out of twenty ST155 isolates (35% of ST155), one out of four ST451 isolates (0.6%) and one out of six ST736 isolates (3.75%) carried plasmids (Supplementary Table S1). All ST155 plasmid carrying isolates were obtained from butter or cow cheese from Milk IND 3. ST451 and ST736 plasmid carrying isolates were obtained from pork meat from unknown and Meat IND 3, respectively.

### 3.4 Antimicrobial genes, pathogenicity islands, and virulence genes

The *fosX* and *lin* genes were detected in all 160 *L. monocytogenes* isolates included in this study.

All known Listeria VGs were detected in the Montenegrin isolates (Supplementary Table S1). Eight out of 160 *L. monocytogenes* isolates (5% of the total) carried pathogenicity island LIPI-3. Specifically, four ST1 (2.5% of the total) isolates, one ST3 (0.6%) isolate, one ST4 (0.6%) isolate and two ST489 (1.25%) isolates obtained from seven different industries carried LIPI-3. Two out of 160 isolates carried LIPI-4 (1.25% of the total). Specifically, one ST4 (0.6%) isolate and one ST517 (0.6%) isolate obtained from different type of meat and different industries carried LIPI-4. One ST4 (0.6%) isolate obtained from beef meat from Meat IND 1 carried both, LIPI-3 and LIPI-4. *L. monocytogenes* ST1 isolates lacked the *actA*, *ami*, *aut*, *gtcA*, *inlF*, *inlJ*, *inlK*, and *vip*. *L. monocytogenes* ST2 lacked the *ami*, *aut*, *gtcA*, *inlF*, *inlJ*, *inlK*, *lapB*, and *vip*. *L. monocytogenes* ST7, ST37, ST204 and ST321 lacked *vip*. *L. monocytogenes* ST8, ST9, ST155, ST451 and ST580 had the complete spectra of VGs. One ST101 isolate lacked VGs *aut*, *fbpA*, *iap/cwhA*, *inlF*, *inlA*, *inlJ*, *inlP*, *lplA1*, *plcB*, and *prfA*, all other ST101 isolates carried all known VGs. *L. monocytogenes* ST121 lacked *actA*, *iap/cwhA*, *inlF*, and *inlJ*.

Briefly, one hundred eighteen out of 160 *L. monocytogenes* isolates carried *InlF,* one hundred twenty *L. monocytogenes* isolates carried *InlJ,* one hundred twenty-five *L. monocytogenes* isolates carried *ActA*, one hundred forty-five *L. monocytogenes* isolates carried *aut*, one hundred forty-six isolates carried *ami* and *InlK*, one hundred fifty-one *L. monocytogenes* isolates carried *GtcA,* one hundred fifty-two *L. monocytogenes* isolates carried *lapB*, one hundred fifty-seven *L. monocytogenes* isolates carried *InlA*, *InlB and InlP*, one hundred fifty-eight *L. monocytogenes* isolates carried *PdgA* and *LplA1*, one hundred fifty-nine *L. monocytogenes* isolates carried *FbpA*, *Hyl*, *plcA*, *plcB, PrfA* and *Bsh*, one hundred sixty *L. monocytogenes* isolates carried *OatA* and *LntA*. *ClpC*, *ClpE*, *ClpP*, *InlC, lap, Mpl, LspA*, *LpeA*, *PrsA2*, and *Iap* genes were present in all *L. monocytogenes* isolates.

## 4 Discussion

### 4.1 Genetic diversity

Controlling *L. monocytogenes* in the food chain is an ongoing and important challenge due to its resilience in different environments. Over a period of 8 years, samples from food products and food production environments of different origin and composition were examined, showing a prevalence rate of 0.7% among all samples analysed. In a similar study conducted in the USA from the period 2005–2017, the prevalence of *L. monocytogenes* in 150,000 RTE meat samples was 0.4% (Mamber et al., 2020), which is similar to the prevalence in RTE meat samples in our study (0.6%). The incidence of *L. monocytogenes* in RTE pork in our study (69%) is significantly higher compared to similar studies (Gamboa-Marin et al., 2012; Wang et al., 2018), where percentages ranged from 13.5–33.9%. The presence of *L. monocytogenes* in RTE milk products was 12%, which is consistent with findings from other studies (Chen et al., 2020). The occurrence in RTE fish was 0.5%, which is lower than reported in recent studies (Korsak et al., 2012; European Food Safety Authority, and European Centre for Disease Prevention and Control, 2017).

In our study, all isolates belonged either to lineage I or II, both of which are known sources of listeriosis outbreaks, as previously described (Fretz et al., 2010; Orsi et al., 2011; Ruppitsch et al., 2015; Gelbíčová et al., 2018; Pietzka et al., 2019; Matle et al., 2020; Benjamin et al., 2023). Genetic characterization of *L. monocytogenes* isolates from Montenegro is crucial for microbiological safety because both, lineage I and lineage II *L. monocytogenes* strains pose a serious threat to public health.

Based on the WGS characterization, the 160 isolates of our study were grouped into four serogroups (IIa, IIb, IIc, and IVb), being serogroup IIa the most prevalent (66.4%), followed by serogroup IIc (21.7%), serogroup IIb (6.2%) and lastly, serogroup IVb (5.6%). Similar findings were reported by Korsak et al. (2012), where 471 *L. monocytogenes* strains isolated from different foods had the following serogroup distribution: IIa (54.4%), IIc (25.5%), IIb (12.5%) and IVb (7.6%). Serogroup IIa was isolated from a wide variety of milk and meat products from several industries mainly from Montenegro, but also from other countries such as France, Spain, Serbia, and Bosnia. These results are in concordance with previous studies characterizing *L. monocytogenes* from different food sources, which showed the higher prevalence of serogroup IIa in comparison to others (Clémentine et al., 2016; Pyz-Lukasik et al., 2022). Some of the characteristics that may contribute to a higher prevalence of serogroup IIa in the food and food processing environment are a more efficient biofilm formation and predomination in biofilms of mixed cultures (Youwen et al., 2009), a greater resistance to bacteriocins (Korsak et al., 2012) and the possession of a great number of plasmids (60% of *L. monocytogenes* serogroup IIa isolates of our study carried at least one plasmid) that confer resistance to toxic metals and possibly other compounds found in the environment (Orsi et al., 2011). Nevertheless, not only group IIa is a contaminant in food since previous studies also reported that these four groups (groups IIa, IIb, IIc, and IVb) are involved in listeriosis worldwide (Gorski, 2021). For instance, the study carried out by Espinosa-Mata et al. (2022) reported that serogroup IVb was the most prevalent among *L. monocytogenes* isolates coming from cheese, which is in the opposite direction from our study since our isolates coming from cheese belongs mainly to serogroup IIa.

The most prevalent STs among our isolates were ST8, ST9, ST121 and ST155, which is in concordance with other studies (Cabal et al., 2019; Shen et al., 2022). In our study, we observed the spreading of the same ST in different meat and dairy industries during different years. ST9 is a common food associated (Althaus et al., 2014; Martín et al., 2014; Maury et al., 2016, 2019) one in most European countries and worldwide (Bespalova et al., 2021). ST9 has been shown to persist in the same facility for more than 9 years successfully overcoming hygienic barriers within the factory (Annette et al., 2020). A possible hypothesis for the occurrence of the exact same ST9 clone in the two industries in Montenegro is that the strain entered the facilities with the raw meat imported from Spain (personal communication).

Although there are no reports on cases of human listeriosis in Montenegro, the fact that hypervirulent ST8 strains are among the most prevalent STs in food samples in this study is of concern. ST8 isolates were detected in almost all meat and dairy industries. A previous study showed that in Germany, thirteen out of thirty-nine listeriosis cases caused by *L. monocytogenes* ST8 were detected at health-care facilities where patients had consumed ready-to-eat meat products from the same manufacturer (Lachmann et al., 2021). *L. monocytogenes* ST8 and ST121 are described to contain plasmids that contribute to a higher tolerance to high temperature, salinity, acidic environments, oxidative stress and disinfectants (Naditz et al., 2019), contributing to their ubiquity and persistence capabilities. These results agree with ours, since all ST8 and ST121 carried plasmids. In our study ST121 is predominantly associated with the meat food industry, which does not contradict other studies where ST121 is predominantly associated with food plant environments (Schmitz-Esser et al., 2015). The *L. monocytogenes* ST155 isolates examined in this study were isolated from milk products and chicken meat from Montenegro and Italy, respectively, as well as from an unknown source from Serbia. Previous studies have documented the involvement of ST155 in clinical cases (Zhao et al., 2021) and outbreaks (Stessl et al., 2022) in recent years. Other STs reported as hypervirulent, such as ST1, ST2, and ST4 were detected in nine isolates coming mainly from meat products but one isolate was derived from cowmilk cheese. Detection of the hypervirulent ST1, ST2 and ST4 strains (Guidi et al., 2021) in our study represents a public health concern. Our ST1 strains differed by a minimum of 40 alleles to ST1 strains from recent studies (Maury et al., 2016; Guidi et al., 2021). National surveillance data from France and the Netherlands indicate this clonal complex as predominant in systemic infections in humans with neurological forms of listeriosis (Maury et al., 2016; Koopmans et al., 2017).

In our study ST101 strains were detected in products from two different industries (Milk IND 1 and Meat IND 2), located within the same industrial yard and being part of the same production brand. While the production procedures of these two industries are separated, our findings suggest that cross-contamination of products with this ST101 strain consistently present in the environment is likely occurring. Recognizing the environment as a key source of *L. monocytogenes*, Linke et al. (2014), underscored the significance of the environment as a primary source of *L. monocytogenes*, as shown in their two-year study of soil and water samples, which revealed ST101 as one of the dominant strains (Linke et al., 2014). In the Listeria SEQuencing (LiSEQ) project ST101 isolates were linked with milk/milk products (Painset et al., 2019). The detection of two identical ST7/CT16794 isolates in 2017, one from fresh cow’s milk from Montenegro and the other from sour cream from Bosnia might be explained by the import of milk contaminated with listeria from Bosnia and Serbia (personal communication). Along with the identification of ST7 strains in clinical and food samples, it has been linked to listeriosis cases in cattle and sheep (Annette et al., 2020; Zhao et al., 2021). Among the 20 different STs isolated from meat products ST7 has been reported, along with others like ST1, ST2, ST3 and ST155 to persist in industrial plants for extended periods. The consistent isolation of isolates with no allelic differences from spatially distant industries suggests a possible contamination originating from the raw material. Notably, the only isolate (ST37) detected in frozen vegetables during the entire testing period was detected in a product imported from France in 2020. This same ST was also found in isolates coming from pork from various meat industries and in cow milk cheese. In addition to a frequent isolation from clinical samples, ST37 has been isolated from meat, fish as well as dairy products (Alvarez-Molina et al., 2021; Kubicová et al., 2021; Maćkiw et al., 2021). ST37 has also been isolated from mallard, pheasant and teal feces, moose carcass and wild boar (Fredriksson-Ahoma et al., 2022). In Austria in 2017, ST37 was the fifth most frequent ST in the category food including meat, dairy products, and vegetables (Cabal et al., 2019).

Several *L. monocytogenes* types of our study were found to be closely related to *L. monocytogenes* strains several other European countries (den Bakker et al., 2013; Maury et al., 2016; Moura et al., 2016, 2024). This highlights the broader European scenario of *L. monocytogenes* dissemination and the conservation of isolation sources across different countries, which is influenced by the international trade of food. These findings underscore the importance of stringent control measures for *L. monocytogenes* strains in the global food chain to prevent cross-border contamination and ensure food safety.

### 4.2 Virulence genes and pathogenicity islands of *Listeria monocytogenes* isolates

LIPIs are specific regions in the genome of *L. monocytogenes* that encode virulence factors playing a crucial role for its pathogenicity (Quereda et al., 2021; Wiktorczyk-Kapischke et al., 2023). Those virulence factors, as previously said, are key factors in the adaptation and spread of *L. monocytogenes* in the environment (Disson et al., 2021; Quereda et al., 2021; Fredriksson-Ahoma et al., 2022). de Melo Tavares et al. (2020) showed that *L. monocytogenes* lineage II did not carry LIPI-3, which is in contrast with our study since three *L. monocytogenes* lineage II in our study carried LIPI-3, although the LIPI-3 carrying *L. monocytogenes* were in the majority lineage IVb. Shen et al. (2022) and Lake et al. (2021) showed that *L. monocytogenes* CC4 was a carrier of LIPI-4, which is in accordance with our findings. However, in our study the other strain with LIPI-4 was *L. monocytogenes* ST517, for which there is no information whether it normally has LIPI-4. Furthermore, numerous studies agree that *L. monocytogenes* ST9 and ST121 do not carry pathogenicity islands of any kind (Shen et al., 2022), which also agrees with our results, since none of them carried neither LIPI-3 nor LIPI-4. Recently, Zhang et al. (2024) described *L. monocytogenes* isolates from RTE carrying LIPI-3 and LIPI-4, as well as ST3 isolates carrying LIPI-3, which is in concordance with the results of our study.

A special feature of *L. monocytogenes* lineage II is the presence of *vip*, *inlF* and *inlK* which has been described only to be present in this lineage and not in others (Pyz-Lukasik et al., 2022), which is in concordance with our results since, for instance all our *L. monocytogenes* lineage IV isolates were missing these genes (*vip*, *inlF*, *inlK*) in addition to the lack of others such as *inlJ*, *gtcA*, *ami*, and *aut*.

Several studies showed that *aut* is not present in *L. monocytogenes* lineage IVb (Cabanes et al., 2004) which agrees with our findings since in all of them *aut* gene was absent. On the other hand, the study carried out by Pyz-Lukasik et al. (2022) suggests that *vip* gene is only present in pathogenic *Listeria* species, while it is absent in non-pathogenic *Listeria*. Some studies revealed that CC9 *L. monocytogenes* is highly associated with the presence of *vip* gen (Wang et al., 2019) which agree with our results (present in eighty-seven isolates). In contrast, some studies reported *L. monocytogenes* ST9 strains (which is one of the STs included in CC9) as hypo virulent. This would contrast with the above and with our results since, if *L. monocytogenes* ST9 is hypo virulent it should have no association with *vip* and instead in our study they do.

### 4.3 Antimicrobial resistance genes, plasmids, and mobile genetic elements

All our isolates carried *lin* and *fosX* ARGs, which is in concordance with other studies (Olaimat, 2018; Hanes and Huang, 2022), *lin* and *fosX* are considered as natural/ intrinsic resistance factors of *L. monocytogenes* conferring resistance to lincosamides and fosfomycin (Olaimat, 2018; Fredriksson-Ahoma et al., 2022; Hanes and Huang, 2022). No acquired ARGs were detected.

Previous studies reported that the most prevalent MGEs found in *L. monocytogenes* were *ISLmo3*, *ISLmo5*, *ISLmo7*, *ISLmo9*, *ISLmo8*, *ISS1N*, *cn_8625_ISS1N* and *cn_12410_ISS1N* (Parra-Flores et al., 2022), which agrees with our findings since all were found in our strains. Tn5422 is a natural transposon of *L. monocytogenes* which can generate deletions being probably the reason for the size diversity of the *L. monocytogenes* plasmids (Lebrun et al., 1994). On the other hand, Tn6188 is structurally related to Tn554 from *Staphylococcus aureus*, which suggests a common origin or horizontal gene transfer within these both species (Müller et al., 2013). In the same study they found that this Tn6188 was present in some *Listeria monocytogenes* strains coming from food and food processing environments predominantly from serovar 1/2a. These results are in concordance with our findings.

ST1, ST2, ST3, ST155 and ST204 have been described as plasmid-carriers clones coming from food (Wagner et al., 2020; Mafuna et al., 2021; Schmitz-Esser et al., 2021). In contrast, in our study *L. monocytogenes* ST1, ST2, ST3, ST155 and ST204 strains did not carry any plasmids. On the other hand, ST101, ST124, ST489 were not yet reported in the literature as plasmid carriers, which is in concordance with our results.

## 5 Conclusion

Monitoring the presence of *L. monocytogenes* over a longer period is a reliable indicator of this pathogen’s opportunities in the food chain. Our results reveal a high genetic diversity and variability of *L. monocytogenes* in the Montenegrin food chain, underscoring the importance of ongoing surveillance. This data is crucial for improving and ameliorating the public health and food safety sector. Whole-genome sequencing (WGS) allows researchers the tracking of the trajectory and persistence of *L. monocytogenes* among other pathogens, and the identification of genetic markers of resistance and virulence. The combination of WGS and seeking the epidemiological link provides a comprehensive view of the pathogen’s spread and impact. Furthermore, the Austrian reference database for *L. monocytogenes*, alongside other extensive and continuously updated pathogen databases, has allowed us to verify that many of our isolates share a close genetic relationship with isolates of the same sequence type (ST) widely distributed across different European countries. These findings reinforce the relevance of our study in a broader European context, not only at the level of Montenegro, establishing relevant connections in the field of food safety and public health.

## Data availability statement

The datasets presented in this study can be found in online repositories. The names of the repository/repositories and accession number(s) can be found in the article/Supplementary material.

## Author contributions

BD: Conceptualization, Data curation, Formal analysis, Investigation, Methodology, Software, Validation, Visualization, Writing – original draft, Writing – review & editing. AP: Conceptualization, Data curation, Formal analysis, Funding acquisition, Investigation, Methodology, Project administration, Resources, Software, Validation, Visualization, Writing – original draft, Writing – review & editing. AM: Conceptualization, Formal analysis, Funding acquisition, Investigation, Resources, Supervision, Visualization, Writing – review & editing. WR: Conceptualization, Formal analysis, Funding acquisition, Investigation, Resources, Software, Supervision, Writing – original draft, Writing – review & editing. IZ: Conceptualization, Data curation, Formal analysis, Investigation, Methodology, Project administration, Resources, Supervision, Validation, Visualization, Writing – original draft, Writing – review & editing.

## Footnotes

^1^ https://bigsdb.pasteur.fr/cgi-bin/bigsdb/bigsdb.pl?db=pubmlst_listeria_isolates&page=profiles

## References

[ref1] Althaus D. Lehner A. Brisse S. Maury M. Tasara T. Stephan R. (2014). Characterization of *Listeria monocytogenes* strains isolated during 2011–2013 from human infections in Switzerland. Foodborne Pathog. Dis. 11, 753–758. doi: 10.1089/fpd.2014.1747, PMID: 25007293

[ref2] Alvarez-Molina A. Cobo-Díaz J. F. López M. Prieto M. de Toro M. Alvarez-Ordóñez A. (2021). Unraveling the emergence and population diversity of *Listeria monocytogenes* in a newly built meat facility through whole genome sequencing. Int. J. Food Microbiol. 340:109043. doi: 10.1016/j.ijfoodmicro.2021.109043, PMID: 33454520

[ref3] Annette F. Solveig L. Trond M. (2020). In-depth longitudinal study of *Listeria monocytogenes* ST9 isolates from the meat processing industry: resolving diversity and transmission patterns using whole-genome sequencing. Appl. Environ. Microbiol. 86, e00579–e00520. doi: 10.1128/AEM.00579-20, PMID: 32414794 PMC7357480

[ref4] Bankevich A. Nurk S. Antipov D. Gurevich A. A. Dvorkin M. Kulikov A. S.. (2012). SPAdes: a new genome assembly algorithm and its applications to single-cell sequencing. J. Comput. Biol. 19, 455–477. doi: 10.1089/cmb.2012.0021, PMID: 22506599 PMC3342519

[ref5] Benjamin F. Karine C. Sandrine T. Arnaud F. Guillaume G. Carole F.. (2023). Identification by high-throughput real-time PCR of 30 major circulating *Listeria monocytogenes* clonal complexes in Europe. Microbiol. Spectr. 11:e0395422. doi: 10.1128/spectrum.03954-22, PMID: 37158749 PMC10269651

[ref6] Bespalova T. Y. Mikhaleva T. V. Meshcheryakova N. Y. Kustikova O. V. Matovic K. Dmitric M.. (2021). Novel sequence types of *Listeria monocytogenes* of different origin obtained in the republic of Serbia. Microorganisms 9:1289. doi: 10.3390/microorganisms9061289, PMID: 34204786 PMC8231576

[ref7] Cabal A. Pietzka A. Huhulescu S. Allerberger F. Ruppitsch W. Schmid D. (2019). Isolate-based surveillance of *Listeria monocytogenes* by whole genome sequencing in Austria. Front. Microbiol. 10:2282. doi: 10.3389/fmicb.2019.02282, PMID: 31632381 PMC6779813

[ref8] Cabanes D. Dussurget O. Dehoux P. Cossart P. (2004). Auto, a surface associated autolysin of *Listeria monocytogenes* required for entry into eukaryotic cells and virulence. Mol. Microbiol. 51, 1601–1614. doi: 10.1111/j.1365-2958.2003.03945.x 15009888

[ref9] Chen Y. Chen M. Wang J. Wu Q. Cheng J. Zhang J.. (2020). Heterogeneity, characteristics, and public health implications of *Listeria monocytogenes* in ready-to-eat foods and pasteurized Milk in China. Front. Microbiol. 11:642. doi: 10.3389/fmicb.2020.00642, PMID: 32351479 PMC7174501

[ref10] Chlebicz A. Slizewska K. (2018). Campylobacteriosis, salmonellosis, Yersiniosis, and Listeriosis as zoonotic foodborne diseases: a review. Int. J. Environ. Res. 15:863. doi: 10.3390/ijerph15050863 PMC5981902 29701663

[ref11] Clémentine H. Benjamin F. Laurent G. Pimlapas L. Damien M. Jean-François M.. (2016). Population genetic structure of *Listeria monocytogenes* strains as determined by pulsed-field gel electrophoresis and multilocus sequence typing. Appl. Environ. Microbiol. 82, 5720–5728. doi: 10.1128/AEM.00583-16, PMID: 27235443 PMC5007763

[ref12] de Melo Tavares R. da Silva D. A. Camargo A. C. Yamatogi R. S. Nero L. A. (2020). Interference of the acid stress on the expression of llsX by *Listeria monocytogenes* pathogenic island 3 (LIPI-3) variants. Food Res. Int. 132:109063. doi: 10.1016/j.foodres.2020.109063 32331684

[ref13] den Bakker H. C. Desjardins C. A. Griggs A. D. Peters J. E. Zeng Q. Young S. K.. (2013). Evolutionary dynamics of the accessory genome of *Listeria monocytogenes*. PLoS One 8:e67511. doi: 10.1371/journal.pone.0067511, PMID: 23825666 PMC3692452

[ref14] Disson O. Moura A. Lecuit M. (2021). Making sense of the biodiversity and virulence of *Listeria monocytogenes*. Trends Microbiol. 29, 811–822. doi: 10.1016/j.tim.2021.01.008 33583696

[ref15] Espinosa-Mata E. Mejía L. Villacís J. E. Alban V. Zapata S. (2022). Detection and genotyping of *Listeria monocytogenes* in artisanal soft cheeses from Ecuador. Rev. Argent Microbiol. 54, 53–56. doi: 10.1016/j.ram.2021.02.013 33906777

[ref16] European Food Safety Authority, and European Centre for Disease Prevention and Control (2017). The European Union summary report on trends and sources of zoonoses, zoonotic agents and food-borne outbreaks in 2016. EFSA J. 15:e05077. doi: 10.2903/j.efsa.2017.5077, PMID: 32625371 PMC7009962

[ref17] Ferreira V. Wiedmann M. Teixeira P. Stasiewicz M. J. (2014). *Listeria monocytogenes* persistence in food-associated environments: epidemiology, strain characteristics, and implications for public health. J. Food Prot. 77, 150–170. doi: 10.4315/0362-028X.JFP-13-150, PMID: 24406014

[ref18] Fredriksson-Ahoma M. Sauvala M. Kurittu P. Heljanko V. Heikinheimo A. Paulsen P. (2022). Characterisation of *Listeria monocytogenes* isolates from hunted game and game meat from Finland. Food Secur. 11:3679. doi: 10.3390/foods11223679 PMC9689155 36429271

[ref19] Fretz R. Pichler J. Sagel U. Much P. Ruppitsch W. Pietzka A. T.. (2010). Update: multinational listeriosis outbreak due to “Quargel”, a sour milk curd cheese, caused by two different *L. monocytogenes* serotype 1/2a strains, 2009–2010. Eur. Secur. 15:19543. doi: 10.2807/ese.15.16.19543-en 20430003

[ref20] Gambarin P. Magnabosco C. Losio M. N. Pavoni E. Gattuso A. Arcangeli G.. (2012). *Listeria monocytogenes* in ready-to-eat seafood and potential hazards for the consumers. Int. J. Microbiol. 2012:497635. doi: 10.1155/2012/497635, PMID: 22761621 PMC3384907

[ref21] Gamboa-Marin A. Buitrago M. S. Perey-Perey K. Mercado R. M. Poutou Pinales R. Carrascal-Camacho A. (2012). Prevalence of *Listeria monocytogenes* in pork-meat and other processed products from the Colombian swine industry. Rev. MVZ Cordoba 17, 2827–2833. doi: 10.21897/rmvz.250

[ref22] Gelbíčová T. Zobaníková M. Tomáštíková Z. Van Walle I. Ruppitsch W. Karpíšková R. (2018). An outbreak of listeriosis linked to Turkey meat products in the Czech Republic, 2012–2016. Epidemiol. Infect. 146, 1407–1412. doi: 10.1017/S0950268818001565, PMID: 29909819 PMC9133684

[ref23] Gorski L. (2021). “Serotype assignment by Sero-agglutination, ELISA, and PCR” in *Listeria Monocytogenes*: methods and protocols. eds. Fox E. M. Bierne H. Stessl B. (New York, NY: Springer US), 57–78. 10.1007/978-1-0716-0982-8_5 32975766

[ref24] Guidi F. Orsini M. Chiaverini A. Torresi M. Centorame P. Acciari V. A.. (2021). Hypo- and hyper-virulent *Listeria monocytogenes* clones persisting in two different food processing plants of Central Italy. Microorganisms 9:376. doi: 10.3390/microorganisms9020376, PMID: 33668440 PMC7918772

[ref25] Hanes R. M. Huang Z. (2022). Investigation of antimicrobial resistance genes in *Listeria monocytogenes* from 2010 through to 2021. Int. J. Environ. Res. Public Health 19:5506. doi: 10.3390/ijerph19095506, PMID: 35564901 PMC9099560

[ref26] Hernandez-Milian A. Payeras-Cifre A. (2014). What is new in Listeriosis? Biomed. Res. Int. 2014:358051. doi: 10.1155/2014/358051, PMID: 24822197 PMC4005144

[ref27] ISO 11290-1:2017. (2022). Horizontal method for the detection and enumeration of Listeria monocytogenes and of Listeria spp. International Organization for Standardization. Available at https://www.iso.org/standard/60313.html

[ref28] Johansson M. H. K. Bortolaia V. Tansirichaiya S. Aarestrup F. M. Roberts A. P. Petersen T. N. (2021). Detection of mobile genetic elements associated with antibiotic resistance in *Salmonella enterica* using a newly developed web tool: MobileElementFinder. J. Antimicrob. Chemother. 76, 101–109. doi: 10.1093/jac/dkaa390, PMID: 33009809 PMC7729385

[ref29] Kimura B. (2006). Recent advances in the study of the genotypic diversity and ecology of *Listeria monocytogenes*. Microbes Environ. 21, 69–77. doi: 10.1264/jsme2.21.69

[ref30] Koopmans M. M. Bijlsma M. W. Brouwer M. C. van de Beek D. van der Ende A. (2017). *Listeria monocytogenes* meningitis in the Netherlands, 1985–2014: a nationwide surveillance study. J. Infect. 75, 12–19. doi: 10.1016/j.jinf.2017.04.004, PMID: 28419853 PMC5513958

[ref31] Korsak D. Borek A. Daniluk S. Grabowska A. Pappelbaum K. (2012). Antimicrobial susceptibilities of *Listeria monocytogenes* strains isolated from food and food processing environment in Poland. Int. J. Food Microbiol. 158, 203–208. doi: 10.1016/j.ijfoodmicro.2012.07.016, PMID: 22874767

[ref32] Kubicová Z. Roussel S. Félix B. Cabanová L. (2021). Genomic diversity of *Listeria monocytogenes* isolates from Slovakia (2010 to 2020). Front. Microbiol. 12:12. doi: 10.3389/fmicb.2021.729050, PMID: 34795648 PMC8593459

[ref33] Lachmann R. Halbedel S. Adler M. Becker N. Allerberger F. Holzer A.. (2021). Nationwide outbreak of invasive listeriosis associated with consumption of meat products in health care facilities, Germany, 2014–2019. Clin. Microbiol. Infect. 27, 1035.e1–1035.e5. doi: 10.1016/j.cmi.2020.09.020, PMID: 32979571

[ref34] Lake F. B. van Overbeek L. S. Baars J. J. P. Koomen J. Abee T. den Besten H. M. W. (2021). Genomic characteristics of *Listeria monocytogenes* isolated during mushroom (Agaricus bisporus) production and processing. Int. J. Food Microbiol. 360:109438. doi: 10.1016/j.ijfoodmicro.2021.109438, PMID: 34715483

[ref35] Lakicevic B. Nastasijevic I. (2016). Food reviews international *Listeria monocytogenes* in retail establishments: contamination routes and control strategies *Listeria monocytogenes* in retail establishments: contamination routes and control strategies. Food Rev. Intl. 33, 247–269. doi: 10.1080/87559129.2016.1175017

[ref36] Lebrun M. Audurier A. Cossart P. (1994). Plasmid-borne cadmium resistance genes in *Listeria monocytogenes* are present on Tn5422, a novel transposon closely related to Tn917. J. Bacteriol. 176, 3049–3061. doi: 10.1128/jb.176.10.3049-3061.1994, PMID: 8188606 PMC205463

[ref37] Lee S. Ward T. J. Graves L. M. Wolf L. A. Sperry K. Siletzky R. M.. (2012). Atypical *Listeria monocytogenes* serotype 4b strains harboring a lineage II-specific gene cassette. Appl. Environ. Microbiol. 78, 660–667. doi: 10.1128/AEM.06378-11, PMID: 22138999 PMC3264116

[ref38] Leong D. NicAogáin K. Luque-Sastre L. McManamon O. Hunt K. Alvarez-Ordóñez A.. (2017). A 3-year multi-food study of the presence and persistence of *Listeria monocytogenes* in 54 small food businesses in Ireland. Int. J. Food. Microbiol. 249, 18–26. doi: 10.1016/j.ijfoodmicro.2017.02.015, PMID: 28271853

[ref39] Linke K. Rückerl I. Brugger K. Karpiskova R. Walland J. Muri-Klinger S.. (2014). Reservoirs of Listeria species in three environmental ecosystems. Appl. Environ. Microbiol. 80, 5583–5592. doi: 10.1128/AEM.01018-14, PMID: 25002422 PMC4178586

[ref40] Liu S. Graham J. E. Bigelow L. Morse P. D. Wilkinson B. J. (2002). Identification of *Listeria monocytogenes* genes expressed in response to growth at low temperature. Appl. Environ. Microbiol. 68, 1697–1705. doi: 10.1128/AEM.68.4.1697-1705.2002 11916687 PMC123842

[ref41] Liu B. Zheng D. Zhou S. Chen L. Yang J. (2022). VFDB 2022: a general classification scheme for bacterial virulence factors. Nucleic Acids Res. 50, D912–D917. doi: 10.1093/nar/gkab1107, PMID: 34850947 PMC8728188

[ref42] Maćkiw E. Korsak D. Kowalska J. Felix B. Stasiak M. Kucharek K.. (2021). Genetic diversity of *Listeria monocytogenes* isolated from ready-to-eat food products in retail in Poland. Int. J. Food Microbiol. 358:109397. doi: 10.1016/j.ijfoodmicro.2021.109397, PMID: 34536853

[ref43] Mafuna T. Matle I. Magwedere K. Pierneef R. E. Reva O. N. (2021). Whole genome-based characterization of *Listeria monocytogenes* isolates recovered from the food chain in South Africa. Front. Microbiol. 12:12. doi: 10.3389/fmicb.2021.669287, PMID: 34276601 PMC8283694

[ref44] Mamber S. W. Mohr T. B. Leathers C. Mbandi E. Bronstein P. A. Barlow K.. (2020). Occurrence of *Listeria monocytogenes* in ready-to-eat meat and poultry product verification testing Samples from U.S. Department of Agriculture–regulated producing establishments, 2005 through 2017. J. Food Prot. 83, 1598–1606. doi: 10.4315/JFP-20-010, PMID: 32324844

[ref45] Martín B. Perich A. Gómez D. Yangüela J. Rodríguez A. Garriga M.. (2014). Diversity and distribution of *Listeria monocytogenes* in meat processing plants. Food Microbiol. 44, 119–127. doi: 10.1016/j.fm.2014.05.014, PMID: 25084653

[ref46] Mateus T. Silva J. Maia R. L. Teixeira P. (2013). Listeriosis during pregnancy: a public health concern. ISRN Obstet. Gynecol. 2013:851712. doi: 10.1155/2013/851712, PMID: 24191199 PMC3804396

[ref47] Matle I. Mbatha K. R. Madoroba E. (2020). A review of *Listeria monocytogenes* from meat and meat products: epidemiology, virulence factors, antimicrobial resistance and diagnosis. Onderstepoort J. Vet. Res. 87:1869. doi: 10.4102/ojvr.v87i1.1869 33054262 PMC7565150

[ref48] Maung A. T. Abdelaziz M. N. S. Mohammadi T. N. Zhao J. EI-Telbany M. Nakayama M.. (2023). Comparison of prevalence, characterization, antimicrobial resistance and pathogenicity of foodborne *Listeria monocytogenes* in recent 5 years in Japan. Microb. Pathog. 183:106333. doi: 10.1016/j.micpath.2023.106333, PMID: 37673352

[ref49] Maury M. M. Bracq-Dieye H. Huang L. Vales G. Lavina M. Thouvenot P.. (2019). Hypervirulent *Listeria monocytogenes* clones’ adaption to mammalian gut accounts for their association with dairy products. Nat. Commun. 10:2488. doi: 10.1038/s41467-019-10380-0, PMID: 31171794 PMC6554400

[ref50] Maury M. M. Tsai Y. H. Charlier C. Touchon M. Chenal-Francisque V. Leclercq A.. (2016). Uncovering *Listeria monocytogenes* hypervirulence by harnessing its biodiversity. Nat. Genet. 48, 308–313. doi: 10.1038/ng.3501, PMID: 26829754 PMC4768348

[ref51] Michael F. Vyacheslav B. HD H. PA B. SD J. Igor T.. (2019). Validating the AMRFinder tool and resistance gene database by using antimicrobial resistance genotype-phenotype correlations in a collection of isolates. Antimicrob. Agents Chemother. 63:e00483-19. doi: 10.1128/aac.00483-19, PMID: 31427293 PMC6811410

[ref52] Michel D. Carmen B. Philippe G. Christine J. Paul M. (2004). Differentiation of the major *Listeria monocytogenes* Serovars by multiplex PCR. J. Clin. Microbiol. 42, 3819–3822. doi: 10.1128/jcm.42.8.3819-3822.2004, PMID: 15297538 PMC497638

[ref53] Moura A. Criscuolo A. Pouseele H. Maury M. M. Leclercq A. Tarr C.. (2016). Whole genome-based population biology and epidemiological surveillance of *Listeria monocytogenes*. Nat. Microbiol. 2:16185. doi: 10.1038/nmicrobiol.2016.185, PMID: 27723724 PMC8903085

[ref54] Moura A. Leclercq A. Vales G. Tessaud-Rita N. Bracq-Dieye H. Thouvenot P.. (2024). Phenotypic and genotypic antimicrobial resistance of *Listeria monocytogenes*: an observational study in France. Lancet Reg. Health Eur. 37:100800. doi: 10.1016/j.lanepe.2023.100800, PMID: 38362545 PMC10866989

[ref55] Müller A. Rychli K. Muhterem-Uyar M. Zaiser A. Stessl B. Guinane C. M.. (2013). Tn6188 – a novel transposon in *Listeria monocytogenes* responsible for tolerance to Benzalkonium chloride. PLoS One 8:e76835. doi: 10.1371/journal.pone.0076835, PMID: 24098567 PMC3788773

[ref56] Naditz A. L. Dzieciol M. Wagner M. Schmitz-Esser S. (2019). Plasmids contribute to food processing environment–associated stress survival in three *Listeria monocytogenes* ST121, ST8, and ST5 strains. Int. J. Food Microbiol. 299, 39–46. doi: 10.1016/j.ijfoodmicro.2019.03.016, PMID: 30953994

[ref57] Olaimat A. (2018). Emergence of antibiotic resistance in *Listeria monocytogenes* isolated from food products: a comprehensive review. Compr. Rev. Food Sci. Food Saf. 17, 1277–1292. doi: 10.1111/1541-4337.12387 33350166

[ref58] Oloketuyi S. F. Khan F. (2017). Inhibition strategies of *Listeria monocytogenes* biofilms—current knowledge and future outlooks. J. Basic Microbiol. 57, 728–743. doi: 10.1002/jobm.201700071, PMID: 28594071

[ref59] Orsi R. H. Den Bakker H. C. Wiedmann M. (2011). *Listeria monocytogenes* lineages: genomics, evolution, ecology, and phenotypic characteristics. Int. J. Med. Microbiol. 301, 79–96. doi: 10.1016/j.ijmm.2010.05.002, PMID: 20708964

[ref60] Painset A. Björkman J. T. Kiil K. Guillier L. Mariet J. F. Félix B.. (2019). LiSEQ – whole-genome sequencing of a cross-sectional survey of *Listeria monocytogenes* in ready-to-eat foods and human clinical cases in Europe. Microb. Genom. 5:e000257. doi: 10.1099/mgen.0.000257 30775964 PMC6421348

[ref61] Parra-Flores J. Holý O. Bustamante F. Lepuschitz S. Pietzka A. Contreras-Fernández A.. (2022). Virulence and antibiotic resistance genes in *Listeria monocytogenes* strains isolated from ready-to-eat foods in Chile. Front. Microbiol. 12:796040. doi: 10.3389/fmicb.2021.796040, PMID: 35299835 PMC8921925

[ref62] Pietzka A. Allerberger F. Murer A. Lennkh A. Stöger A. Cabal Rosel A.. (2019). Whole genome sequencing based surveillance of *L. monocytogenes* for early detection and investigations of Listeriosis outbreaks. Front. Public Health 7:139. doi: 10.3389/fpubh.2019.00139, PMID: 31214559 PMC6557975

[ref63] Poyart-Salmeron C. Carlier C. Trieu-Cuot P. Courvalin P. Courtieu A. L. (1990). Transferable plasmid-mediated antibiotic resistance in *Listeria monocytogenes*. Lancet 335, 1422–1426. doi: 10.1016/0140-6736(90)91447-I, PMID: 1972210

[ref64] Pyz-Lukasik R. Paszkiewicz W. Kielbus M. Ziomek M. Gondek M. Domaradzki P.. (2022). Genetic diversity and potential virulence of *Listeria monocytogenes* isolates originating from polish artisanal cheeses. Food Secur. 11:2805. doi: 10.3390/foods11182805, PMID: 36140933 PMC9497517

[ref65] Quereda J. J. Morón-García A. Palacios-Gorba C. Dessaux C. García-del Portillo F. Pucciarelli M. G.. (2021). Pathogenicity and virulence of *Listeria monocytogenes*: a trip from environmental to medical microbiology. Virulence 12, 2509–2545. doi: 10.1080/21505594.2021.1975526, PMID: 34612177 PMC8496543

[ref66] Ragon M. Wirth T. Hollandt F. Lavenir R. Lecuit M. Le Monnier A.. (2008). A new perspective on *Listeria monocytogenes* evolution. PLoS Pathog. 4:e1000146. doi: 10.1371/journal.ppat.1000146, PMID: 18773117 PMC2518857

[ref67] Robertson J. Nash J. H. E. (2018). MOB-suite: software tools for clustering, reconstruction and typing of plasmids from draft assemblies. Microb. Genom. 4:e000206. doi: 10.1099/mgen.0.000206 30052170 PMC6159552

[ref68] Ronholm J. (2018). Editorial: game changer – next generation sequencing and its impact on food microbiology. Front. Microbiol. 9:363. doi: 10.3389/fmicb.2018.00363, PMID: 29593663 PMC5854679

[ref69] Ruppitsch W. Prager R. Halbedel S. Hyden P. Pietzka A. Huhulescu S.. (2015). Ongoing outbreak of invasive listeriosis, Germany, 2012 to 2015. Euro Surveill. 20:30094. doi: 10.2807/1560-7917.ES.2015.20.50.30094 26691727

[ref70] Salcedo C. Arreaza L. Alcalá B. de la Fuente L. Vázquez J. A. (2003). Development of a multilocus sequence typing method for analysis of *Listeria monocytogenes* clones. J. Clin. Microbiol. 41, 757–762. doi: 10.1128/jcm.41.2.757-762.2003, PMID: 12574278 PMC149676

[ref71] Schmitz-Esser S. Anast J. M. Cortes B. W. (2021). A large-scale sequencing-based survey of plasmids in *Listeria monocytogenes* reveals global dissemination of plasmids. Front. Microbiol. 12:653155. doi: 10.3389/fmicb.2021.653155, PMID: 33776982 PMC7994336

[ref72] Schmitz-Esser S. Müller A. Stessl B. Wagner M. (2015). Genomes of sequence type 121 *Listeria monocytogenes* strains harbor highly conserved plasmids and prophages. Front. Microbiol. 6:380. doi: 10.3389/fmicb.2015.00380, PMID: 25972859 PMC4412001

[ref73] Shen J. Zhang G. Yang J. Zhao L. Jiang Y. Guo D.. (2022). Prevalence, antibiotic resistance, and molecular epidemiology of *Listeria monocytogenes* isolated from imported foods in China during 2018 to 2020. Int. J. Food Microbiol. 382:109916. doi: 10.1016/j.ijfoodmicro.2022.109916, PMID: 36126498

[ref74] Stessl B. Ruppitsch W. Wagner M. (2022). *Listeria monocytogenes* post-outbreak management – when could a food production be considered under control again? Int. J. Food Microbiol. 379:109844. doi: 10.1016/j.ijfoodmicro.2022.109844, PMID: 35985077

[ref75] Wagner E. Zaiser A. Leitner R. Quijada N. M. Pracser N. Pietzka A.. (2020). Virulence characterization and comparative genomics of *Listeria monocytogenes* sequence type 155 strains. BMC Genomics 21:847. doi: 10.1186/s12864-020-07263-w, PMID: 33256601 PMC7708227

[ref76] Wang Y. Li X. Osmundson T. Shi L. Yan H. (2019). Comparative genomic analysis of a multidrug-resistant *Listeria monocytogenes* ST477 isolate. Foodborne Pathog. Dis. 16, 604–615. doi: 10.1089/fpd.2018.2611 31094569

[ref77] Wang H. Luo L. Zhang Z. Deng J. Wang Y. Miao Y.. (2018). Prevalence and molecular characteristics of *Listeria monocytogenes* in cooked products and its comparison with isolates from listeriosis cases. Front. Med. 12, 104–112. doi: 10.1007/s11684-017-0593-9, PMID: 29372499

[ref78] Werner R. Ariane P. Karola P. Stefan B. Lasa F. H. Franz A.. (2015). Defining and evaluating a Core genome multilocus sequence typing scheme for whole-genome sequence-based typing of *Listeria monocytogenes*. J. Clin. Microbiol. 53, 2869–2876. doi: 10.1128/jcm.01193-15, PMID: 26135865 PMC4540939

[ref79] Wiktorczyk-Kapischke N. Skowron K. Wałecka-Zacharska E. (2023). Genomic and pathogenicity islands of *Listeria monocytogenes*—overview of selected aspects. Front. Mol. Biosci. 10:1161486. doi: 10.3389/fmolb.2023.1161486, PMID: 37388250 PMC10300472

[ref80] Youwen P. Frederick B. Sophia K. (2009). Competition of *Listeria monocytogenes* serotype 1/2a and 4b strains in mixed-culture biofilms. Appl. Environ. Microbiol. 75, 5846–5852. doi: 10.1128/AEM.00816-09, PMID: 19648379 PMC2747858

[ref81] Zhang P. Ji L. Wu X. Chen L. Yan W. Dong F. (2024). Prevalence, genotypic characteristics, and antibiotic resistance of *Listeria monocytogenes* from retail foods in Huzhou, China. J. Food Prot. 87:100307. doi: 10.1016/j.jfp.2024.100307, PMID: 38797247

[ref82] Zhao Q. Hu P. Li Q. Zhang S. Li H. Chang J.. (2021). Prevalence and transmission characteristics of Listeria species from ruminants in farm and slaughtering environments in China. Emerg. Microbes Infect. 10, 356–364. doi: 10.1080/22221751.2021.1888658, PMID: 33560938 PMC7928038

[ref83] Zuber I. Lakicevic B. Pietzka A. Milanov D. Djordjevic V. Karabasil N.. (2019). Molecular characterization of *Listeria monocytogenes* isolates from a small-scale meat processor in Montenegro, 2011–2014. Food Microbiol. 79, 116–122. doi: 10.1016/j.fm.2018.12.005, PMID: 30621866

