# Supplementary figures and images for: Surveillance and genetic characterization of Listeria monocytogenes in the food chain in Montenegro during the period 2014–2022
Source: Front Microbiol. 2024 Aug 1;15:1418333. doi: 10.3389/fmicb.2024.1418333 (PMC11324475; doi:10.3389/fmicb.2024.1418333)

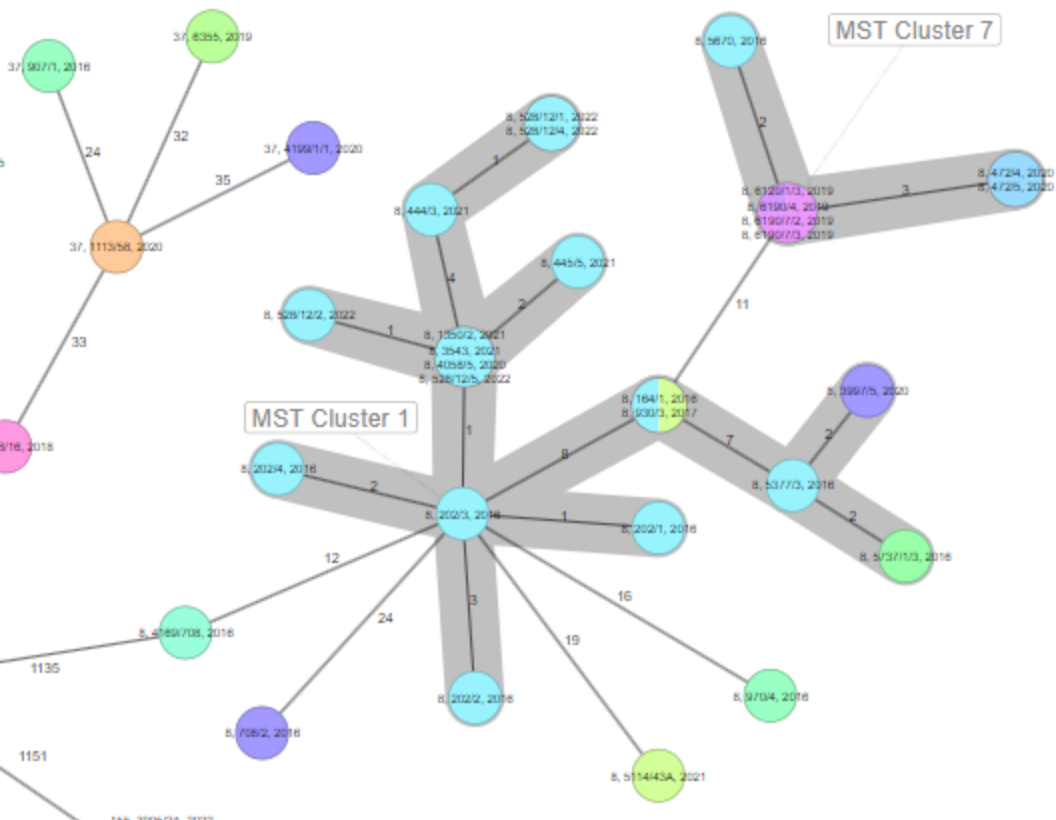

Supplement: Supplementary file 1 [file Data_Sheet_1.PDF]

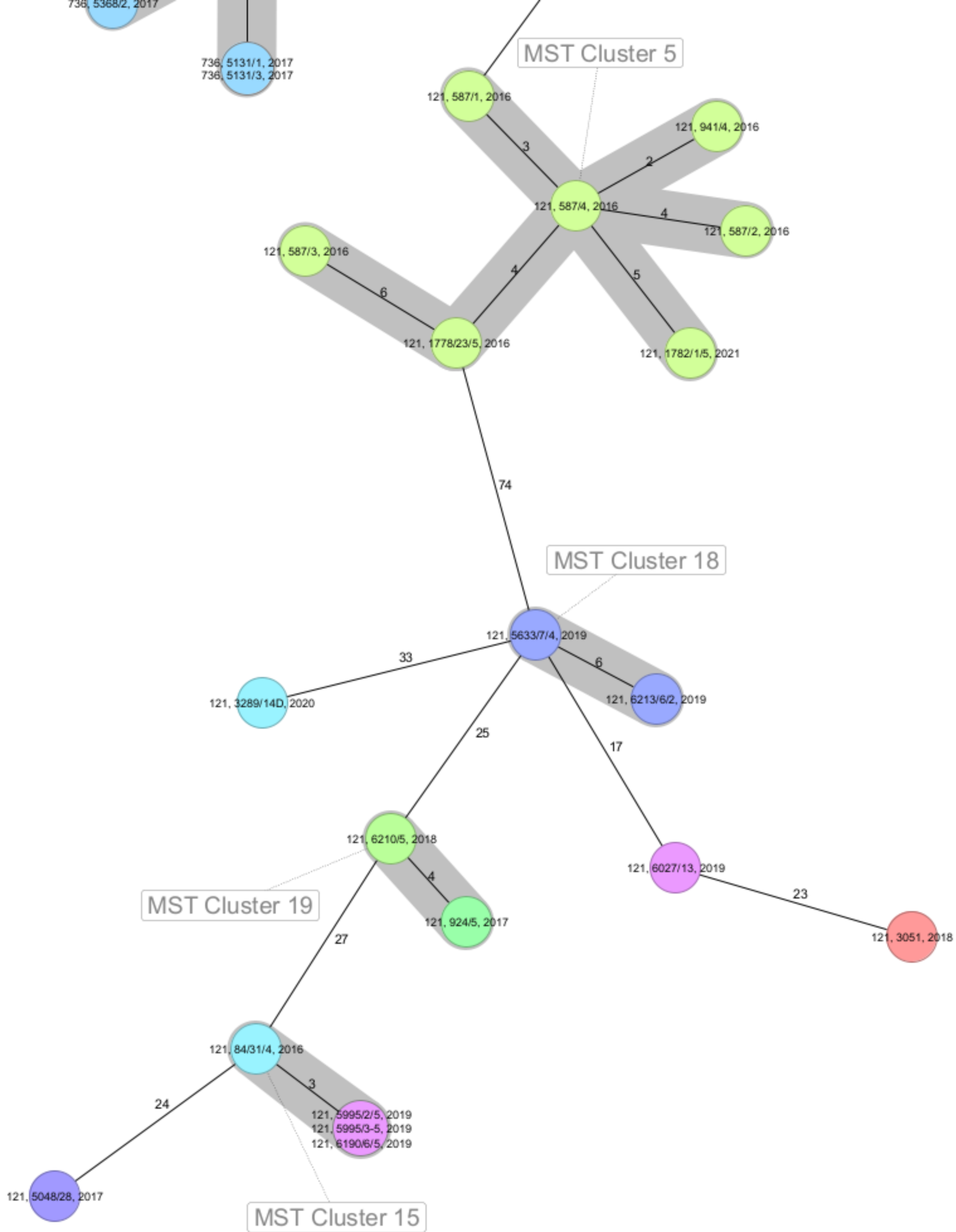

Supplement: Supplementary file 2 [file Data_Sheet_2.PDF]

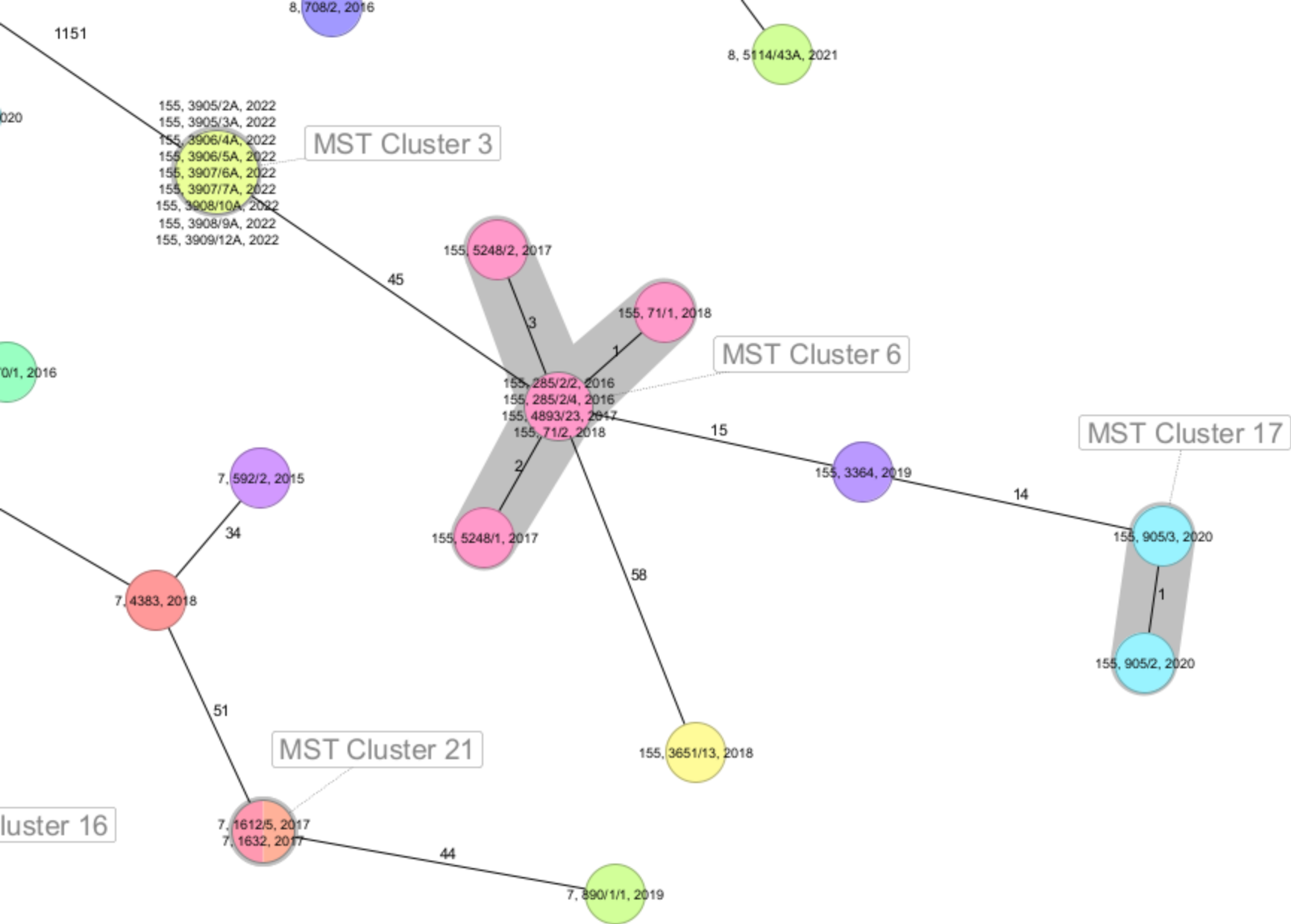

Supplement: Supplementary file 3 [file Data_Sheet_3.PDF]

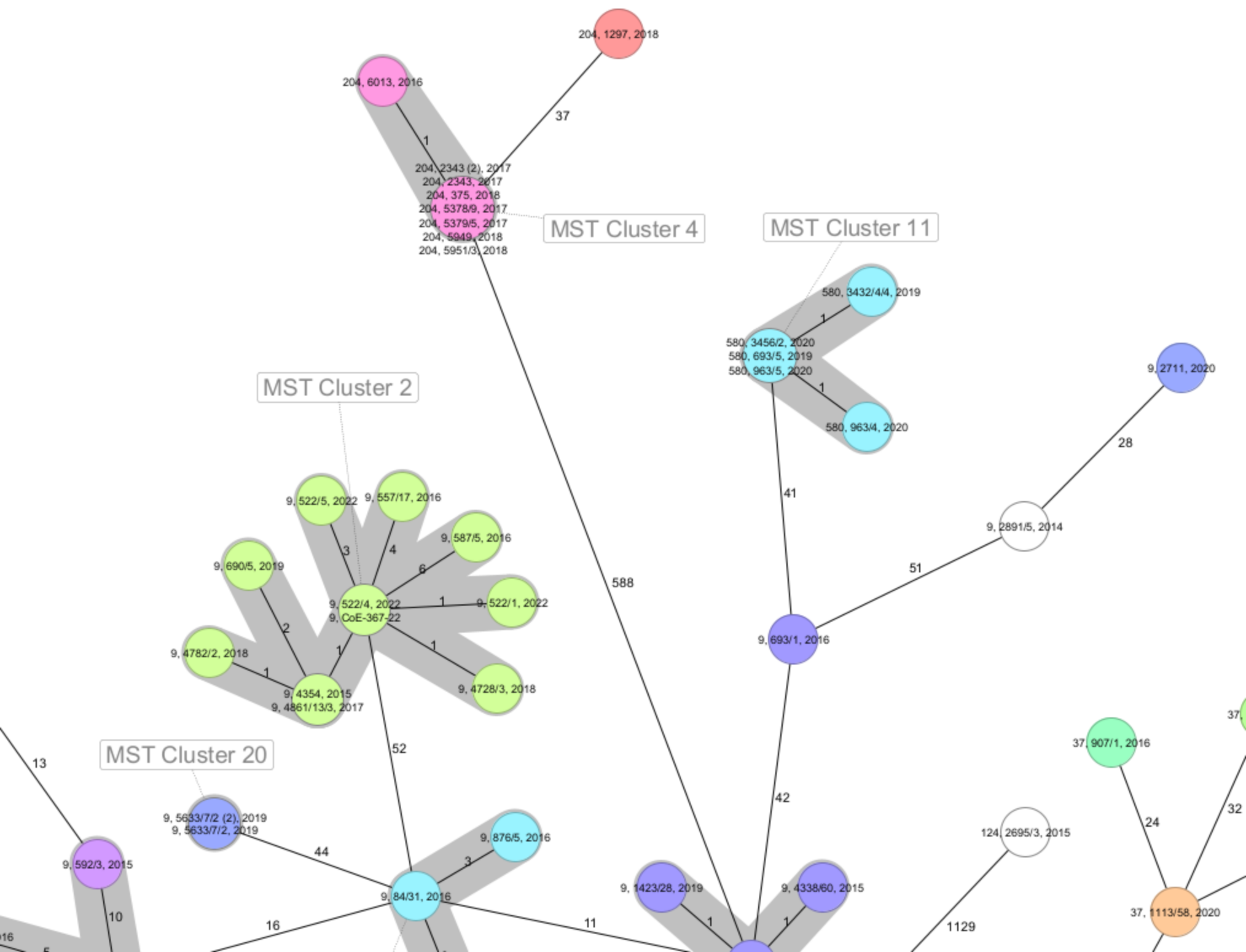

Supplement: Supplementary file 4 [file Data_Sheet_4.PDF]
