# Supplementary material for: Surveillance and genetic characterization of Listeria monocytogenes in the food chain in Montenegro during the period 2014–2022
Source: Front Microbiol. 2024 Aug 1;15:1418333. doi: 10.3389/fmicb.2024.1418333 (PMC11324475; doi:10.3389/fmicb.2024.1418333)
Supplement: Supplementary file 5 [file Data_Sheet_5.PDF]

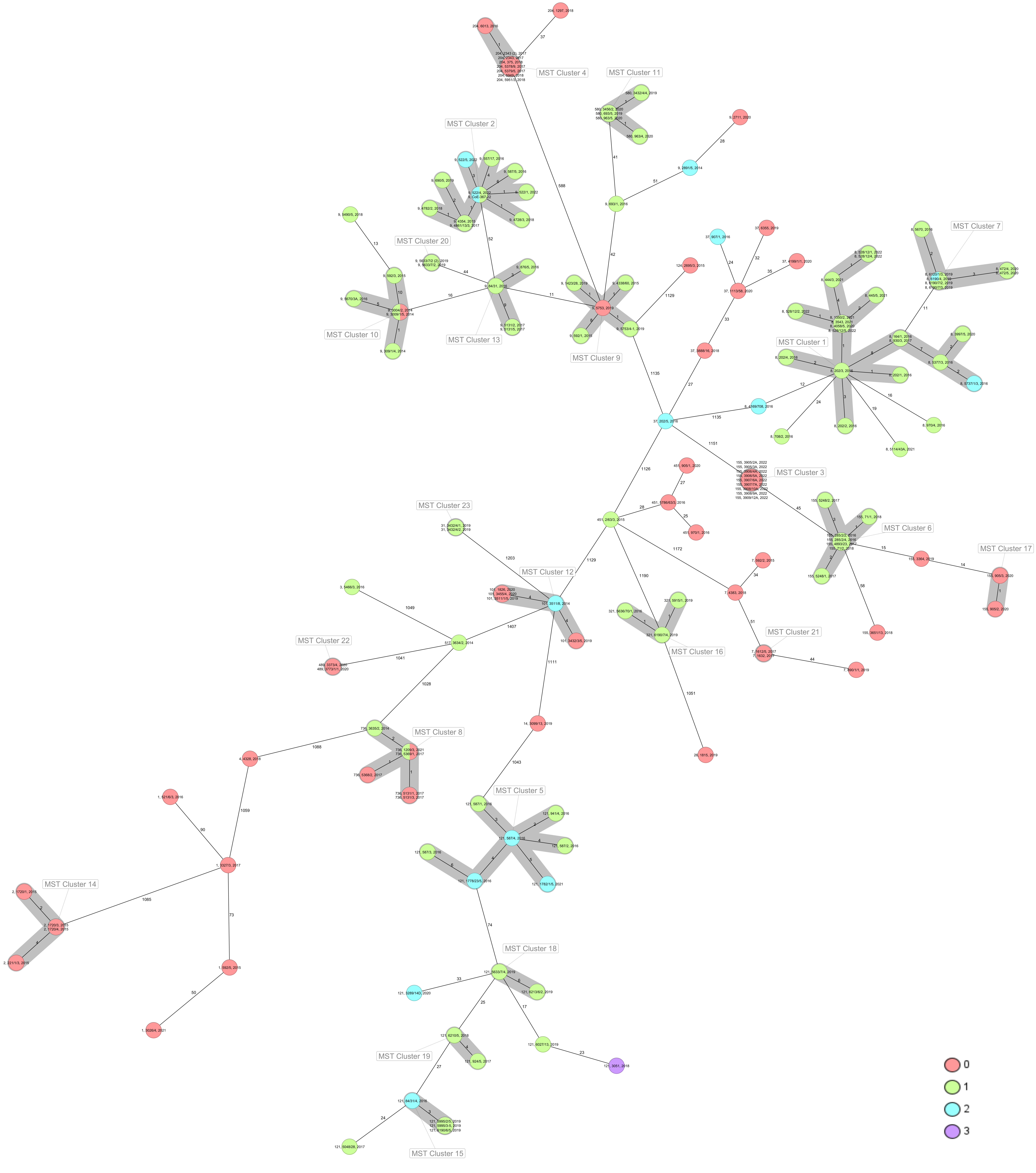

MST Cluster 1
MST Cluster 2
MST Cluster 3
MST Cluster 4
MST Cluster 5
MST Cluster 6
MST Cluster 7
MST Cluster 8
MST Cluster 9
MST Cluster 10
MST Cluster 11
MST Cluster 12
MST Cluster 13
MST Cluster 14
MST Cluster 15
MST Cluster 16
MST Cluster 17
MST Cluster 18
MST Cluster 19
MST Cluster 20
MST Cluster 21
MST Cluster 22
MST Cluster 23
0
1
2
3
